# VNIR–NIR hyperspectral imaging fusion targeting intraoperative brain cancer detection

**DOI:** 10.1038/s41598-021-99220-0

**Published:** 2021-10-04

**Authors:** Raquel Leon, Himar Fabelo, Samuel Ortega, Juan F. Piñeiro, Adam Szolna, Maria Hernandez, Carlos Espino, Aruma J. O’Shanahan, David Carrera, Sara Bisshopp, Coralia Sosa, Mariano Marquez, Jesus Morera, Bernardino Clavo, Gustavo M. Callico

**Affiliations:** 1 Institute for Applied Microelectronics, University of Las Palmas de Gran Canaria, 35017 Las Palmas de Gran Canaria, Spain; 2 Nofima, Norwegian Institute of Food Fisheries and Aquaculture Research, Muninbakken 9-13, Breivika, 6122, NO-9291 Tromsø, Norway; 3 Department of Neurosurgery, Instituto de Investigación Sanitaria de Canarias (IISC), University Hospital Doctor Negrin of Gran Canaria, Barranco de la Ballena S/N, 35010 Las Palmas de Gran Canaria, Spain; 4 Research Unit, Instituto de Investigación Sanitaria de Canarias (IISC), University Hospital Doctor Negrin of Gran Canaria, Barranco de la Ballena S/N, 35010 Las Palmas de Gran Canaria, Spain

**Keywords:** Cancer imaging, CNS cancer, Translational research, Brain imaging, Biomedical engineering, Computational science

## Abstract

Currently, intraoperative guidance tools used for brain tumor resection assistance during surgery have several limitations. Hyperspectral (HS) imaging is arising as a novel imaging technique that could offer new capabilities to delineate brain tumor tissue in surgical-time. However, the HS acquisition systems have some limitations regarding spatial and spectral resolution depending on the spectral range to be captured. Image fusion techniques combine information from different sensors to obtain an HS cube with improved spatial and spectral resolution. This paper describes the contributions to HS image fusion using two push-broom HS cameras, covering the visual and near-infrared (VNIR) [400–1000 nm] and near-infrared (NIR) [900–1700 nm] spectral ranges, which are integrated into an intraoperative HS acquisition system developed to delineate brain tumor tissue during neurosurgical procedures. Both HS images were registered using intensity-based and feature-based techniques with different geometric transformations to perform the HS image fusion, obtaining an HS cube with wide spectral range [435–1638 nm]. Four HS datasets were captured to verify the image registration and the fusion process. Moreover, segmentation and classification methods were evaluated to compare the performance results between the use of the VNIR and NIR data, independently, with respect to the fused data. The results reveal that the proposed methodology for fusing VNIR–NIR data improves the classification results up to 21% of accuracy with respect to the use of each data modality independently, depending on the targeted classification problem.

## Introduction

Brain cancer is the most common central nervous system cancer (> 90%) and it represents an highly relevant source of mortality and morbidity, especially in children^1,2^. It can be divided into primary, if cancer arises in the brain, and secondary or metastasis, if cancer starts elsewhere in the body and has spread to the brain^3^. In the United States, there are 10,000 new cases of primary brain tumors each year, being the peak age of onset between 65 and 69 years old^3^. Primary brain tumors are divided into low-grade and high-grade depending on their malignity. Glioblastoma (grade IV) is the most lethal and most common primary brain tumor (50%) with a 5-year survival rate of 5.5%^4^. In low-grade gliomas (grade II) the early and total resection of the tumor increase the overall survival to a 5-year survival rate of 81% and 50 for oligodendroglioma and diffuse astrocytoma, respectively^4^. In case of non-malignant primary tumors, meningiomas are the most common and their resection can prevent further disease progression. A successful resection of the tumor is associated with prolonged survival. Nonetheless, due to the nature and location of the tumor, the complete resection is not always possible or can produce neurological damages to the patient. Hence, surgeons have to find a balance between tumor removal and neurologic compromise^5^.

The accurate identification of the boundaries between tumor and normal tissue during surgery improves the resection. Currently, neurosurgeons use several intraoperative guidance tools for tumor resection assistance, such as intraoperative Image Guided Stereotactic (IGS) neuronavigation, intraoperative Magnetic Resonance Imaging (iMRI), or fluorescent tumor markers like 5-aminolevulinic acid (5-ALA)^6^. However, these tools present several limitations, for example, iMRI is an expensive procedure due to require specific operation rooms with no ferromagnetic elements, increasing the surgical time^7^. The changes in tumor volume that occurs during craniotomy and the brain shift are not covered in IGS navigation^8^. 5-ALA is only able to identify high-grade gliomas administering orally a contrast agent to the patient, being an invasive methodology that can cause side effects in the patient^9,10^. Therefore, there is a current need to explore new imaging modalities that could overcome such limitations.

Hyperspectral imaging (HSI) is an emerging, non-contact, non-ionizing, label-free, and minimally invasive sensing technology widely employed in many applications, such as remote sensing^11^, food quality assessment^12^, defense and security^13^, among others^14^. Particularly, the use of HSI has been investigated in several medical applications^15^, such as oncology^16^, dermatology^17^, ophthalmology^18^, endoscopy^19^, wound care^20^, cervical cancer^21^, digital and computational pathology^22^, biomarkers discovery and validation^23^, tissue perfusion measurement^24^, gastroenterology^22^, etc. Hyperspectral (HS) images are composed by hundreds of spectral channels, conforming a continuous spectrum in each pixel which allows the differentiation of the materials which are present in the scene based in their chemical composition^25^. HS cameras generally use Charge-Coupled Device (CCD) or Complementary Metal-Oxide Semiconductor (CMOS) sensors to cover the spectral range between 400 and 1000 nm (visual and near infrared–VNIR), while Indium Gallium Arsenide (InGaAS) and Mercury Cadmium Telluride (MCT) sensors are used to cover the range from 900 to 1700 nm (near infrared–NIR) and 900 to 2500 nm (near short-wave infrared–SWIR), respectively^25^. Thus, to obtain a broadband spectral range image, more than one HS camera is required, involving image registration and fusion algorithms to generate a combined HS image.

On one hand, image fusion techniques are used in many applications to merge information from different sensors with the goal of improving the classification or segmentation results^26^. Usually this image fusion procedure is performed using multispectral images, which have low-spatial and high-spectral resolution, combined with panchromatic images, which have high-spatial but low-spectral resolution, to obtain a new fused image with high-spatial and high-spectral resolution^27^. Spectral fusion is applied to combine the spectral information from different sensors aiming to obtain an HS image with a broadband spectral range. This approach has been employed to identify geographical origins of herbal medicines^28^ or to identify metallic alloys from the recycling industry^29^.

On the other hand, image registration techniques have the goal to match two or more images of the same scene obtained by using different sensors or devices. The image registration is a necessary step to correctly perform the image fusion. Image registration methods can be classified into two groups: intensity-based and features-based techniques^30^. The former uses the intensity values of the image to find similarities between the images in the scene to perform the registration. This technique is widely used to register Computerized Tomography (CT), Magnetic Resonance (MR) images with Positron Emission Tomography (PET) images, among other imaging modalities for computer-aided diagnosis, e.g. in brain tumor detection^31,32^. The later uses morphological structures presented in the image to extract points, lines, curves, etc., in order to find similar features in the images and perform the image registration. There are different feature detectors and extractors, including Scale Invariant Feature Transform (SIFT), Features from Accelerated Segment Test (FAST) or Harris detector. All these methods have been widely used in the literature due to they are robust and automatic algorithms to extract features^33^. Features-based technique have been used in fusion information from different sensors or image mosaic technology^34,35^.

Previous works from this research team have involved the use of HSI in the VNIR spectral range for the intraoperative detection of brain tumors in real-time^36–38^. In this work, a new approach is proposed for performing the spectral fusion of two HS cubes obtained with two different HS cameras covering the VNIR and NIR spectral range with the goal of obtaining an HS cube with a broadband spectral range. This fusion procedure is investigated targeting an improvement of the previous results in the processing of the intraoperative HS brain tumors including NIR information.

## Results

### VNIR–NIR spatial registration using the HSI registration dataset

**Figure 1:**
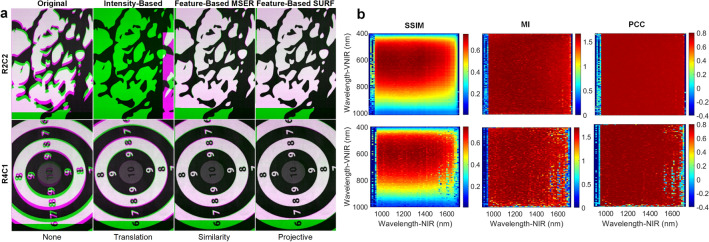
VNIR–NIR Spatial Registration using the *HSI registration dataset*. (**a**) Two registration result examples applying different registration techniques. Both images are overlapped using green-magenta false-color, VNIR (green) and NIR (magenta). First column shows the default registration without applying any type of transformation to the data. Second, third and fourth columns show the results of the intensity-based, feature-based with MSER, and feature-based with SURF techniques, respectively, using the best transformation method. (**b**) Coarse search results of the Structural Similarity Index Measure (SSIM), the Mutual Information (MI), and the Pearson’s Correlation Coefficient (PCC) for identifying the suitable spectral bands for the registration using the feature-based SURF technique with projective transformation.

The *HSI registration dataset* (see Fig. S1a in the Supplementary Material) were registered using different registration techniques and geometric transformations with the purpose of selecting the image registration technique (see “Methods” section) which provides the best result. The VNIR–NIR spatial registration was evaluated computing the structural Similarity Index Measure (SSIM), the Mutual Information (MI), and the Pearson’s Correlation Coefficient (PCC) metrics. In preliminary analysis, a gray-scale image was generated from a pseudo-RGB image of both HS cubes for performing the registration. Figure S2 in the Supplementary Material shows the average SSIM, MI and PCC results obtained after performing the different geometric transformation over the *HSI registration dataset*. In the case of intensity-based technique, translation, similarity, and affine transformations were applied. In the case of feature-based technique using Maximally Stable Extremal Regions (MSER) and Speeded Up Robust Features (SURF) detectors, the transformations employed were affine, similarity and projective. Due to the randomized nature of the M-estimator Sample Consensus (MSAC) algorithm, in the feature-based technique, one thousand consecutive executions were performed to estimate the geometric transformation. The feature-based technique using SURF detector offered the best registration (Fig. S2c in the Supplementary Material). The results obtained using affine and projective transformations were similar. This is produced due to the projective transformation performs the same geometric transform (scaling, shear, rotation, and translation) than the affine transformation, in addition to apply tilt to the transformation. These results outperform the feature-based technique using MSER and also the intensity-based technique. Figure 1a shows two example results of the *HSI registration dataset*, R2C2 and R4C1. The first column shows the registration result without applying any geometric transformation, while the remaining columns show the best results obtained with each registration technique and the best geometric transformation. These images represent an overlay of the VNIR and NIR pseudo-RGB images using green-magenta false-color images. Magenta and green pixels indicate misregistration between the VNIR and NIR images, respectively. The areas with gray-scale pixels indicate areas where the two registered images have similar intensity values. Using the translation transformation in the intensity-based registration, R2C2 is incorrectly registered, while R4C1 improves the registration respect to the result without applying any transformation. These incorrect registrations can be produced due to the random noise that can be found in some spectral bands, affecting to the maximum intensity. The feature-based MSER technique using similarity transformation improves the intensity-based technique but some misregistered pixels can be observed in both images. Finally, the feature-based SURF technique with projective transformation offered the best results. For this reason, this method was selected to be applied in the subsequent experiments. Figure S3 in Supplementary Material shows the remaining registrations of the *HSI registration dataset*.

A coarse-to-fine search was performed using gray-scale images from a single spectral band extracted from both cameras to identify the VNIR and NIR bands, which offer the best registration performance. To reduce the high computational time, the coarse search was performed using steps of seven and three bands in the VNIR and NIR images, respectively, to diminish the number of combinations. Figure 1b shows the R2C2 and R4C1 heatmaps resulting from the coarse search using SSIM, MI and PCC metrics (the remaining heatmaps are shown in Fig. S4 in the Supplementary Material). It can be observed that in all metrics the lower and higher bands for each camera do not offer a correct registration mainly due to the low performance of the sensor in such bands. The MI and PCC metrics indicate all band combinations in the central region offer similar results. In opposite, SSIM metric indicates that regions 500–700 nm and 950–1500 nm in the VNIR and NIR ranges, respectively, achieve the highest results. This is caused because the SSIM metric takes into account the image structure while the other metrics only consider the image intensity. For this reason, to select the optimal spectral bands in the coarse-to-fine search only the SSIM metric was employed. The fine search was performed within the previously selected regions using steps of one band for both cameras. Figure S5 in the Supplementary Material shows the SSIM results using the optimal band combination and summarizes the bands/wavelengths employed. One thousand consecutive executions were performed using the best band combination of each VNIR–NIR HS image pair to obtain the transformation with the highest SSIM value. Finally, the best transformation model was selected after applying each projective transformation to all the images from the *HSI registration dataset*. Figure S6 in the Supplementary Material shows the SSIM boxplot results for each transformation model, where an average SSIM value of ~ 0.78 was obtained for all models. The R2C1 model was selected as it presented the lowest IQR (Interquartile Range). No statistically significant differences were found across the mean SSIM values between R2C1 and R2C2 (which has the higher mean value), using a paired, two-tailed Student’s *t* test at the 5% significance level.

### VNIR–NIR spectral fusion using the HSI spectral reference dataset

**Figure 2:**
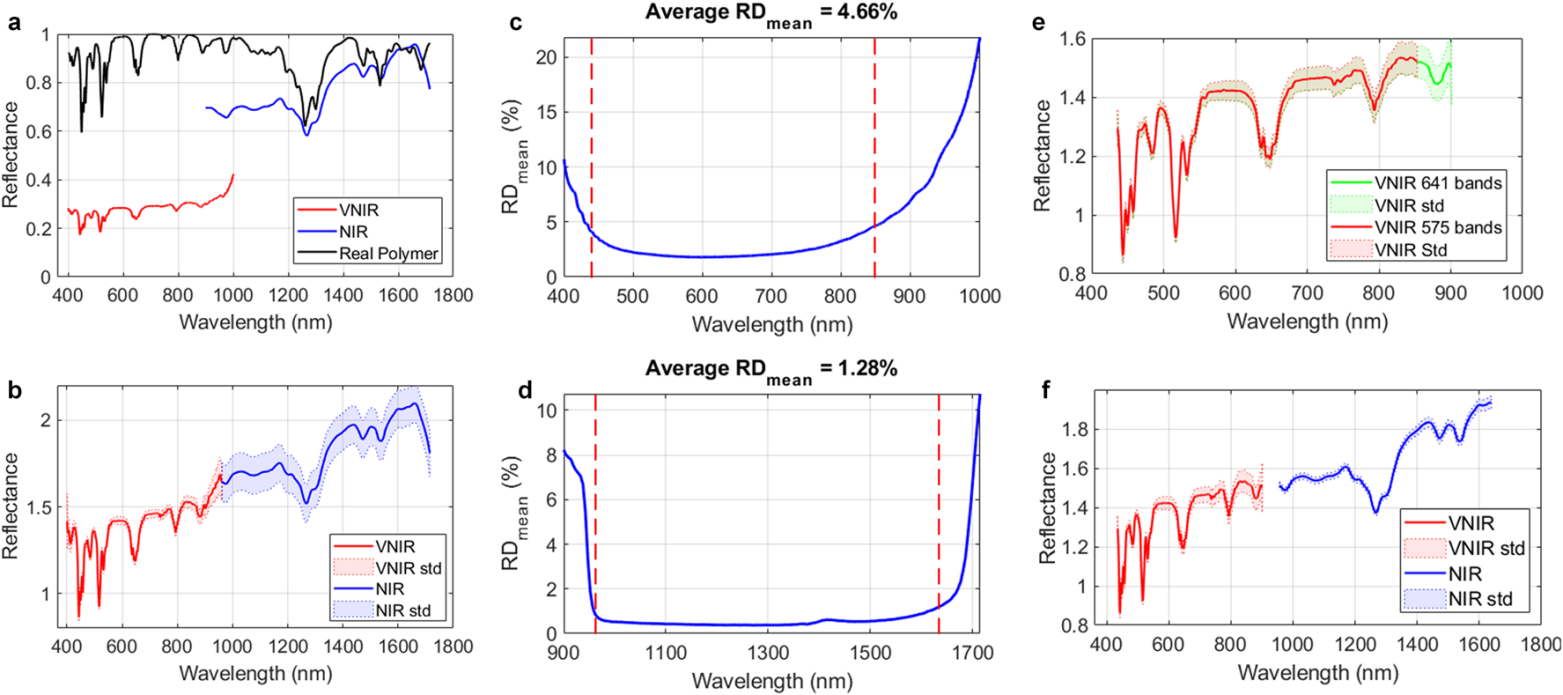
(**a**) Manufactured certified spectral signature of the Zenith Polymer and spectral signatures captured by the VNIR and NIR cameras. (**b**) Fused spectral signature using a common spectral band in the overlapped spectral region between VNIR and NIR data. (**c**, **d**) Average absolute relative difference percentage (RD) results of SR1 using VNIR and NIR data. Red dashed lines represent the initial and final cutoff points for voiding the low performance of the HS sensors. (**e**) Comparison between the mean and std of the Zenith Polymer VNIR spectral signature with 641 spectral bands (green) and 575 spectral bands (red). (f) Fused spectral signature of the Zenith Polymer after applying the proposed VNIR–NIR spectral fusion method.

Considering the low performance of the push-broom HS sensors in the lower and higher spectral bands, a spectral analysis of the data was performed using a *HSI spectral reference dataset* (see Fig. S1b in the Supplementary Material) to evaluate which bands should be removed before performing the spectral fusion. Both HS cameras have a common spectral range between 900 and 1000 nm (Fig. 2a). However, performing a spectral fusion based on the use of this common spectral region is not suitable in this case due to the low performance of the VNIR sensor in those bands. As shown in Fig. 2b, this method causes the NIR region of the fused spectral signature to have a higher standard deviation than the VNIR region when capturing a calibration polymer (see “Methods” section). Hence, a spectral analysis was performed computing the absolute relative difference percentage ($RD$) metric [see Eq. (S13) in the “Methods” section in the Supplementary Material] using the image pairs of each image in the *HSI spectral reference dataset* for both VNIR and NIR cameras. Figure 2c, d shows the $RD_{{mean{ }}}$ values for each wavelength in the VNIR and NIR spectral signatures of a white reference (SR1), respectively. The $RD_{mean}$ represents the average RD value of all pixels in the image at a certain wavelength. In the case of the VNIR data (Fig. 2c), the $RD_{mean}$ is higher than the average from 400 to 435 nm and from 800 to 1000 nm. In the case of the NIR data (Fig. 2d), the $RD_{mean}$ values obtained in the ranges 900–960 nm and 1619–1700 nm are higher than the average. These ranges are represented in the figures using the vertical red dashed lines. The $Average RD_{{mean{ }}}$ value was used to establish the initial and final cutoff point for the selection of the operating bandwidth in each image of the *HSI spectral reference dataset*.

Table S1 and Fig. S7 in the Supplementary Material show the cut off points for each image of the *HSI spectral reference dataset*. It can be observed that the initial cutoff points in the NIR data are the same in the three cases ($\lambda = 956.6 \pm 0 \,{\text{nm}}$), while in the VNIR data there are quite similar values around $\lambda = 435.2 \pm 0.4\, {\text{nm}}$. Considering the final cutoff point, the NIR data values are close to $\lambda = 1632.0 \pm 11.0 \,{\text{nm}}$, while the VNIR data values are close to $\lambda = 849.6 \pm 3.3 \,{\text{nm}}$. In the VNIR case, the final cut off point involves the removal of ~ 200 spectral bands. With the purpose of reducing the number of bands to be removed, an additional analysis was performed using three image pairs from the *HSI plastic dataset* (see Fig. S1c in the Supplementary Material). In this case, the initial cutoff point does not coincide in two of three VNIR image pairs respect to the *HSI spectral reference dataset*, providing an average point of $\lambda = 496.5 \pm 70.1 nm$. This is produced mainly due to the spectral contributions of the plastic color (red and magenta). Considering the final cutoff point in the VNIR data, the average value is higher with respect to the *HSI spectral reference dataset* ($\lambda = 896.0 \pm 14.7\, {\text{nm}}$). In the case of the NIR data, the initial and final cutoff points are similar to the previous ones, $\lambda = 959.8 \pm 2.8 \,{\text{nm}}$ and $\lambda = 1638.4 \pm 9.5 \,{\text{nm}}$, respectively. At this point, a qualitative assessment of the VNIR cutoff points was performed by plotting the mean and standard deviation (std) of the spectral signatures of the Zenith Polymer reflectance standard. Figure 2e shows that the std values between 849 and 900 nm (green) are quite similar to the previous spectral bands (red). For this reason, the selected cutoff points for the VNIR data were 435 and 901 nm, having 641 spectral bands, while the NIR data covered a spectral range between 956 and 1638 nm formed by 144 spectral bands. Finally, the VNIR–NIR spectral fusion was performed applying a reflectance offset to the NIR spectrum in order to adjust the reflectance values of both spectral signatures. The fused spectral signature has a gap between 901 and 956 nm (Fig. 2f), in order to preserve the original standard deviation of the NIR spectrum.

### Evaluation of image segmentation and classification using the HSI plastic dataset

Different unsupervised segmentation and supervised classification techniques were employed to evaluate the performance of the three data types (*VNIR*, *NIR* and *Fused*) in three different segmentation/classification problems: *color*, *material*, and *material-color* identification.

**Figure 3:**
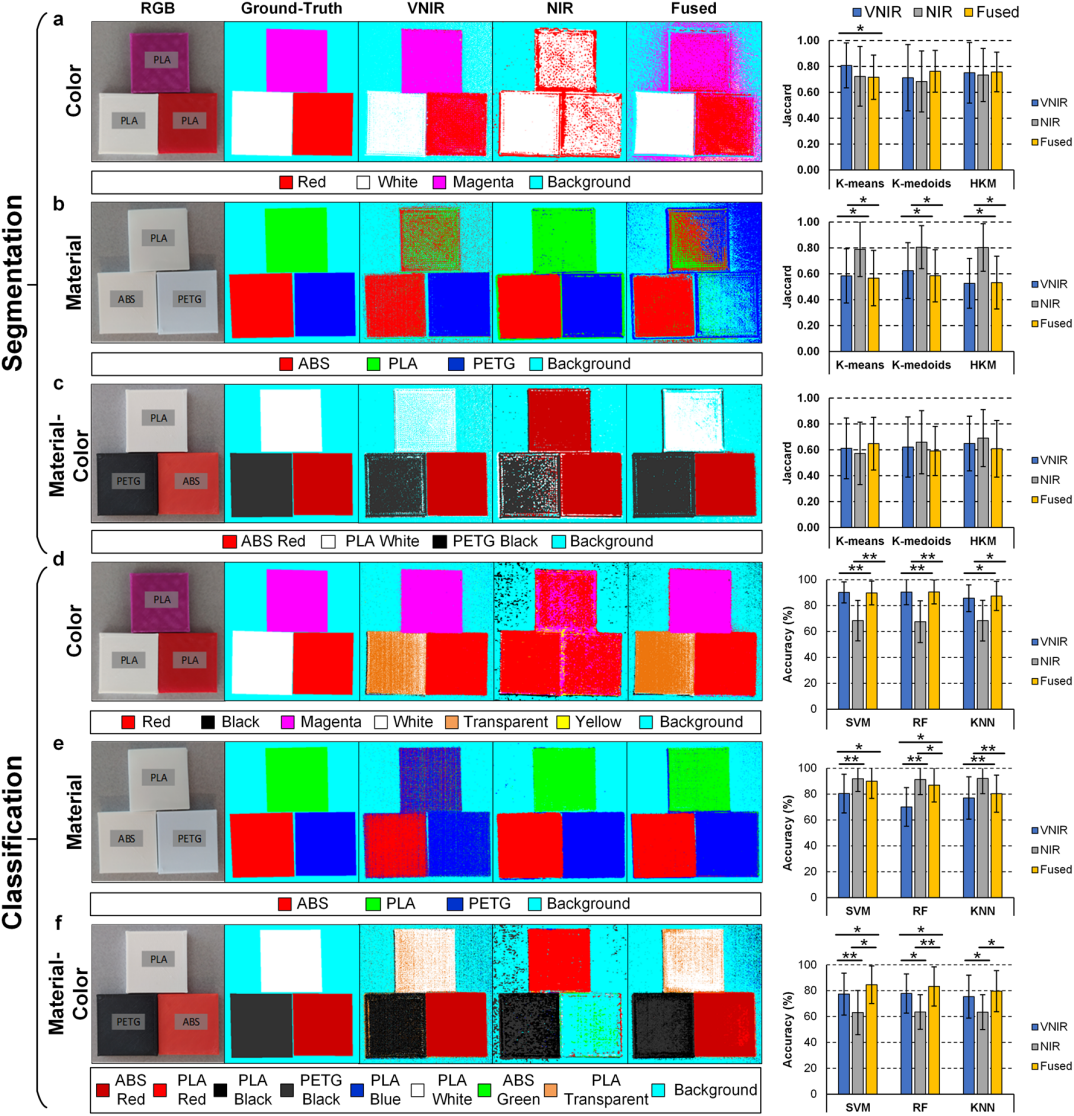
Segmentation and classification maps of three examples of the test set from the *HSI plastic dataset* and average Jaccard and accuracy results obtained from the thirteen images. *Color*, *material*, and *material-color* segmentation (**a**–**c**) and classification (**d**–**f**) problems, respectively, using VNIR, NIR, and fused data. Each column (from left to right) represents the RGB images obtained with a digital camera, the ground-truth maps, the VNIR, NIR, and fused segmentation results, respectively, and the average Jaccard and accuracy results obtained with the entire test set for the three different segmentation and classification algorithms. Results were statistically analyzed using a paired, one-tailed Student’s *t* test at the 5% significance level. (*) Statistically significant difference ($$p < 0.05$$). (**) Highly statistically significant difference ($$p < 0.001)$$. ABS: Acrylonitrile Butadiene Styrene; PLA: Polylactic Acid; PETG: Polyethylene Terephthalate Glycol. HKM: Hierarchical K-means; SVM: Support Vector Machines; RF: Random Forest; KNN: K-Nearest Neighbors.

K-means, K-medoids, and hierarchical K-means algorithms were applied to the test set of *HSI plastic dataset* (see Fig. S1c in the Supplementary Material). Figure 3a–c shows, as examples, the segmentation maps obtained with K-means algorithm from three of the thirteen test HS images, as well as the average Jaccard results obtained with the entire dataset for the three segmentation algorithms. The Jaccard metric was computed using the ground-truth image and the segmentation map of each HS image. As expected, the VNIR data achieved the highest results in the *color* segmentation using K-means algorithm, followed by the Fused data using K-medoids and hierarchical K-means (Fig. 3a), while the *material* identification was superior using the NIR data in all three algorithms (Fig. 3b). However, the *material-color* segmentation of the NIR data using hierarchical K-means improved the segmentation results followed by the Fused data using K-means (Fig. 3c). Statistical analysis was performed to the segmentation results using a paired, one-tailed Student’s *t*-test at 5% significance level. No statistically differences were found between the results of the *material-color* segmentation problem. Tables S2, S3, and S4 in Supplementary Material details the Jaccard results applying K-means, K-medoids, and hierarchical K-means algorithms, respectively, for each test HS image and the average and standard deviation values.

Support Vector Machines (SVMs), Random Forest (RF), and K-Nearest Neighbors (KNN) algorithms were employed for the supervised classification. A coarse-to-fine search (in the case of SVM) and a coarse search (in the case of RF and KNN) were performed to optimize the hyperparameters of each classifier. This procedure was accomplished using the training and validation sets for each data type and classification problem independently. Table S5 and Figs. S8, S9, and S10 in the Supplementary Material shows the optimal hyperparameter values found for each classifier, data type and classification problem, as well as the overall accuracy results obtained in the validation set.

Once SVM, KNN, and RF models were trained and optimized for each case, the classifiers were evaluated using the test set to assess the results obtained in the validation set. Figure 3d–f shows as examples, the classification maps obtained with the SVM classifier from three of the thirteen test HS images, as well as the average overall accuracy results obtained with the entire dataset for the three supervised algorithms. The accuracy was computed using the ground-truth image and the classification map of each HS image. In the *color* classification, VNIR and Fused data, using SVM and RF classifiers, obtained quite similar performance, while NIR data decreases the accuracy in the three classifiers (Fig. 3d). As it can be observed in the SVM example, NIR data misclassifies the three plastic samples, while the VNIR and Fused data identify correctly two out of three samples, misclassifying the white color, which is identified as transparent (orange color in Fig. 3d). On the contrary, in the *material* classification, the NIR data achieved the highest accuracy in all three classifiers, followed by the Fused data using SVM classifier (Fig. 3e). In the *material* example applying SVM classifier, VNIR data only classified two out of three samples correctly, while NIR and Fused data were able to successfully identify the three samples. Finally, in the *material-color* classification the Fused data outperformed the other two data types (Fig. 3f). Statistical analysis was performed to the classification results using a paired, one-tailed Student’s *t* test at 5% significance level. In the material-color classification problem, statistically significant differences were found between the VNIR and Fused data results. Tables S6, S7, and S8 in the Supplementary Material details the accuracy results obtained with SVM, RF, and KNN classifiers for each test HS image and their average and standard deviation values.

### Qualitative evaluation of image segmentation using the *HSI brain dataset*

The proposed VNIR–NIR spectral fusion method was applied to the HS images from the *HSI brain dataset* (Fig. S1d in Supplementary Material). The main goal of this experiment was to evaluate, as a proof-of-concept, if the proposed data fusion method could improve the morphological edge detection of different tissue structures (particularly normal tissue and blood vessels) that can be found in the exposed brain surface during surgery. Image segmentation based on the K-means algorithm was performed in each HS image independently for a qualitative evaluation of the results obtained using the three data types. Quantitative evaluation was not performed due to the low number of pixels labeled in each image, which produced extremely low Jaccard values. The methodology followed to generate this segmentation maps is detailed in the “Methods” sections.

**Figure 4:**
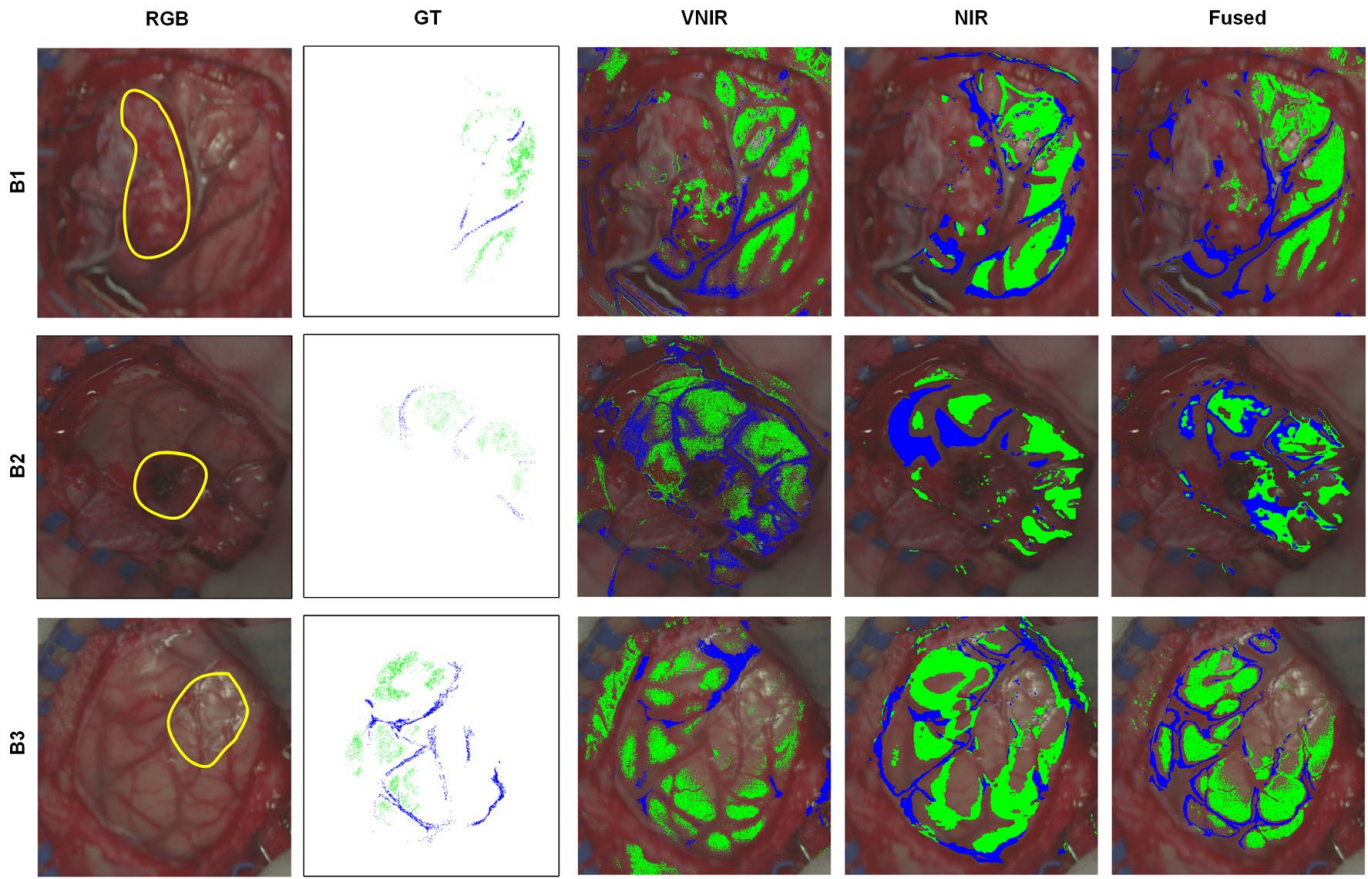
Results of the image segmentation of the *HSI brain dataset*. Each column (from left to right) represents the pseudo-RGB image generated form the VNIR data, the ground-truth map, the VNIR, NIR, and fused segmentation maps overlapped with the pseudo-RGB image, respectively. Green color represents normal tissue and blue color represents blood vessels. B1: Meningioma Grade I; B2: Glioblastoma Grade IV; B3: Glioblastoma Grade IV.

Figure 4 shows the pseudo-RGB images (generated from the VNIR data, where the approximate tumor area has been delineated with a yellow line by visual inspection of the operating surgeon according to the patient’s MRI), the ground-truth maps (green and blue pixels represent normal and blood vessel classes, respectively, and white pixels are non-labelled pixels), and the segmentation maps for the VNIR, NIR, and Fused data overlapped with the pseudo-RGB images. Blue and green colors were selected to be consistent with previous works^36^. Figure S11 in the Supplementary Material shows the average and standard deviation of the spectral signatures of the labeled HSI brain dataset in the different images of the VNIR and NIR data. After a visual evaluation of the segmentation maps by the operating surgeons, it can be observed that in B1, the VNIR map presents normal pixels in the tumor area and normal and blood vessel pixels out of the parenchymal area. In contrast, NIR and fused maps reduce the misclassifications in the tumor area. Moreover, the anatomical structures of the parenchymal area are better defined in the fusion map than in the VNIR and NIR maps, although some pixels are identified as normal within the tumor area. In B2, the VNIR map defines well the anatomical structures of the vessels and normal tissues, while the NIR map avoids misclassifications within the tumor area, delimiting well the parenchyma. The fused map offers a tradeoff between the information shown in the VNIR and NIR maps, but some false negatives are presented in the tumor area. In B3, the tumor area was correctly defined in the VNIR map without false negatives, but the anatomical structures of vessels are not accurately identified. In opposite, the NIR map improve de delineation of blood vessels, but the anatomical structure of normal tissue is poorly defined, including also false negatives in the tumor area. Finally, the fused map offers the best anatomical structures and delineation of tumor area. These results were assessed by the operating surgeons analyzing the MRI of the patient and the pathological diagnosis of the tissue.

## Discussion

Current guidance tools employed to assist brain tumor resection during surgery have several limitations^7–10^. The IGS neuronavigation provide an accurate identification of tumor boundaries in low-grade gliomas, but not in high-grade ones, being affected also by the brain shift phenomenon. To accurately identify high-grade gliomas, it is necessary the use of contrast agents with complex and expensive systems, such as 5-ALA, or employing iMRI devices that requires especial operating rooms and extends the duration of the surgery. Moreover, the choice of the guidance tool to be used in the surgery is determined by the intraoperative pathological result, which may take up to 45 min. Reducing the surgery time implies decreasing the risk of complications during the operation, such as infection, ischemia, respiratory problems, etc., thus improving cost-efficiency. Furthermore, an accurate delimitation between tumor and normal tissue improves the average survival of the patient^5^. For these reasons, it is desirable to develop minimally invasive, label-free and flexible guidance tools that allow identifying brain tumor boundaries in real-time during surgery. The use of HSI in medical applications has been proved to be a valuable resource to identify tumor tissue^16^. Previous works of this research group employed an HS acquisition system composed by VNIR and NIR cameras to capture HS images of in-vivo human brain tissue during surgical procedures with the goal of identifying tumor boundaries in real-time^36–38^. However, in these works only VNIR information was processed due to the impossibility of performing a reliable labeling in the NIR HS images. In this research, this acquisition system has been modified to combine both sources of information (VNIR and NIR) and propose a VNIR–NIR imaging fusion approach to determine, as a proof-of-concept, if the fused data can improve the delimitation of different brain tissue structures with respect to the use of both sources of data independently. In the previous configuration, the VNIR and NIR image registration was not possible to be performed due to the camera non-perpendicularity with respect to the scene, especially in non-flat surface situations (e.g., after tumor resection beginning). Hence, the VNIR labeling could not be used for the NIR images. Additionally, the labeling could not be directly performed over the NIR images due to their low spatial resolution and the false color representation of the pseudo-RGB. The proposed acquisition system configuration allows performing the VNIR–NIR spatial registration, being possible to extrapolate the VNIR labeling to the NIR images and perform a spectral fusion of both sources of data. Additionally, a speedup factor of 2× was achieved in the acquisition time since the capturing is performed in a single scanning.

To achieve an accurate VNIR–NIR spatial registration, several techniques and geometric transformations were analyzed and tested using different HS images. Additionally, a coarse-to-fine search was performed using all the combinations of gray-scale images (extracted from each spectral band) from both HS cameras to identify the most suitable bands for performing the spatial registration. The feature-based technique using SURF detector and projective transformation was selected for the VNIR–NIR spatial registration. Next, a detailed analysis of the VNIR and NIR spectral signatures was performed to determine the optimal operating bandwidth captured by each camera, being combined in the subsequent spectral fusion process. The resulting HS cube was formed by 641 spectral bands in the VNIR range (435–901 nm) and 144 spectral bands in the NIR range (956–1638 nm).

To determine the discrimination capability of the fused data compared with the use of the VNIR and NIR data independently, three segmentation and classification problems were proposed using a controlled HSI dataset based on plastic samples of different materials and colors. The results show that VNIR data identified better the color of the samples than the NIR and fused data, while the material is more accurately identified using the NIR data. However, when the goal is to identify the material and color of the sample, the fused data offered better results than the VNIR and NIR data. Therefore, the selection of the data type to be employed in a certain classification/segmentation problem will be determined by the nature of the materials, substances or tissue to be analyzed. If the optical properties are more relevant in the VNIR region than in the NIR region (or vice versa), then, using the fused data could provide misclassifications in the results. On the contrary, if relevant optical properties can be found in the two spectral ranges (as in the *material-color* problem), the fused data could provide improved discrimination performance.

Finally, a preliminary analysis of three HS images of in-vivo human brain tissue obtained during surgical procedures was performed to evaluate, as a proof-of-concept, the segmentation results generated after processing the three data types. In this preliminary analysis, only two classes (normal and blood vessel) were labeled in the ground-truth maps and employed to reveal the two best clusters associated to such labeled pixels. Analyzing these segmentation results, specialists determined that the Fused maps provided a good tradeoff between the information presented in the VNIR and NIR maps, offering improved anatomical structures delineation. In this experiment, no tumor pixels were labeled or taken into account for the clustering analysis. For such reason, further experiments must be conducted including an increased dataset of HS images from in-vivo brain (where tumor pixels will be also labeled) with the goal of performing both segmentation and classification problems, aiming to identify tumor boundaries and compare the results obtained with the three data types. Moreover, a clinical study, including large number of patients, different tumor types, and performing histological verification of several biopsies (within the tumor area and margins), should be performed to validate the classification results provided by the proposed method.

Additionally, an analysis of the most relevant spectral bands of the fused HS images for an accurate delineation of the tumor boundaries will be explored in future works with the goal of determining the minimum number of wavelengths required to develop customized HS cameras. This will allow a reduction of the acquisition system size and also a time reduction of the data acquisition and processing, targeting real-time performance during surgery. This identification of the most relevant spectral bands in the NIR range will also allow to increase the spatial resolution of this HS images, possibly avoiding the resampling process employed in this work. These advances could allow the development of a novel guidance tool based on HSI technology for the accurate identification of brain tumors, regardless of tumor grade, avoiding the use of several independent devices during surgery and, hence, reducing the operation time.

## Methods

### Processing framework overview

**Figure 5:**
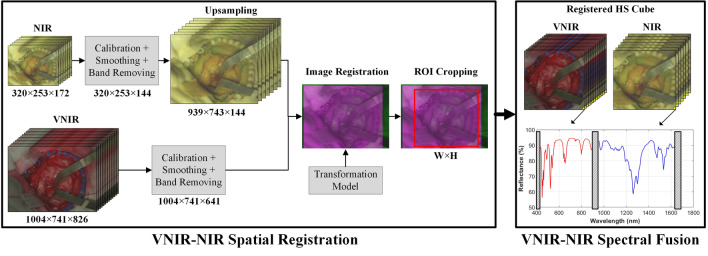
Block diagram of the proposed processing framework based on VNIR–NIR spatial registration combined with spectral fusion. NIR: Near-Infrared; VNIR: Visual and Near-Infrared; ROI: Region of Interest; W: Width; H: Height; HS: Hyperspectral.

The proposed method is composed by two main stages: (1) VNIR–NIR spatial registration; (2) VNIR–NIR spectral fusion (Fig. 5). In the first stage, the VNIR and NIR raw images are pre-processed applying image calibration to avoid the influence of environmental illumination, noise filtering and band removing to reduce the noise in the spectral signatures due to the camera sensor performance (especially in the extreme bands). After that, the NIR image is upsampled to reach the VNIR pixel size, allowing to perform the image registration using a transformation model previously generated. In this transformation the fixed image is the VNIR, and the moving image is the NIR. When both VNIR and NIR images are registered, both images are cropped to obtain the same region of interest (ROI). Finally, in the last stage, the spectra from both VNIR and NIR images are combined, applying a reflectance offset to the NIR spectrum, to perform the spectral fusion and generate a single HS image.

### Hyperspectral acquisition system

**Figure 6:**
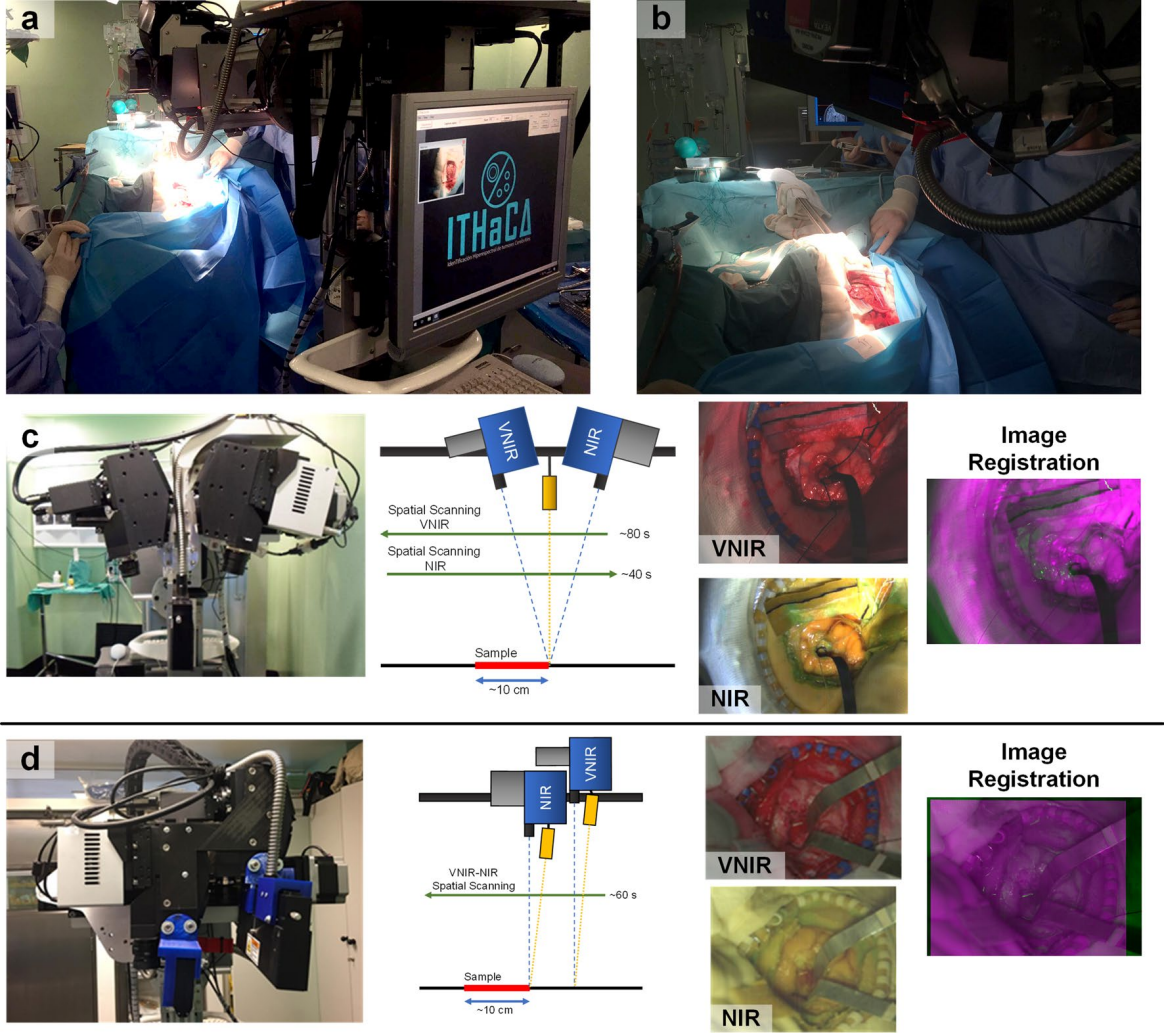
(**a**, **b**) HS acquisition system being used during a neurosurgical intervention at the University Hospital of Gran Canaria Doctor Negrin (Spain). (**c**) HS cameras orientation of the original demonstrator, examples of pseudo-RGB images of VNIR and NIR HS cubes and their corresponding registration using green (VNIR) and magenta (NIR) false color. (**d**) Proposed configuration with HS cameras placed in parallel and perpendicular to the sample, examples of pseudo-RGB images of VNIR and NIR HS cubes and their corresponding registration.

An intraoperative HS demonstrator was developed with the goal of delineating brain tumors during surgical operations (Fig. 6a, b), aiding neurosurgeons during the brain tumor resection^37^. This demonstrator was composed by two push-broom HS cameras (Fig. 6c): the VNIR camera covered the spectral range between 400 and 1000 nm and the NIR between the 900–1700 nm. The illumination system was based on a Quartz Tungsten Halogen (QTH) lamp of 150 W with a broadband emission between 400 and 2200 nm. The light source was connected to a cold light emitter through an optical fiber to avoid the high temperatures of the QTH lamp in the exposed brain surface. The HS cameras and the cold light emitter were installed in a scanning platform to provide the necessary movement for the push-broom technique to generate the complete HS cubes. The working distance between the lens of the cameras and the exposed brain tissue was 40 cm. The field of view (FOV) of both cameras was oriented and aligned to the beam of the cold light emitter to obtain the highest reflectance value in the sensors (Fig. 6c). As a result, both cameras were tilted to capture the same FOV, producing that both HS cubes had different perspectives of the scene and being not possible to achieve an accurate registration for data fusion (Fig. 6c). In this work, different modifications of the acquisition system were performed to obtain the optimal cameras orientation. In the proposed configuration of the acquisition system, both HS cameras are oriented perpendicular to the surface to be captured. Figure 6d shows the position of the cameras in the scanning platform. In this case, it was necessary to include another illumination device, one for each HS camera, and both HS cameras have a similar FOV, allowing an accurate image registration. The working distance between the lens of the cameras and the area to be captured were ~ 33 and ~ 42 cm for the NIR and VNIR cameras, respectively. In addition, the acquisition time of the modified system to capture both HS cubes was reduced to ~ 60 s, performing only a scanning in a single direction. This improvement represented a time reduction of 1 min, due to the original system required ~ 80 and ~ 40 s for the VNIR and NIR HS cubes capturing, respectively, involving two scanning movements in both directions as shown in Fig. 6c.

### Hyperspectral database

The HS database used in this research was divided into four sets to evaluate the different stages of the proposed VNIR–NIR fusion method. The first one was used to evaluate the VNIR–NIR spatial registration (*HSI registration dataset,* Fig. S1a in the Supplementary Material), the second one was employed to validate the VNIR–NIR spectral fusion (*HSI spectral reference dataset,* Fig. S1b in the Supplementary Material), and the last two sets were used to evaluate quantitatively and qualitatively the method performance (*HSI plastic dataset* and *HSI brain dataset,* Fig. S1c, d in the Supplementary Material). The *HSI brain dataset* is formed by three HS images of in vivo brain tissue acquired at the University Hospital of Gran Canaria Doctor Negrin, Spain. Written informed consent was obtained from all the participant subjects, and the study protocol and consent procedures were approved by the *Etica de la Investigacion/Comite de Etica de la Investigacion con Medicamentos (DEI/CEIM)* of the University Hospital Doctor Negrin (2019-001-1). All research was performed in accordance with relevant guidelines/regulations. All four HS datasets were captured using the proposed configuration of the HS acquisition system previously described. The *HSI plastic dataset* the dataset was organized into three groups for performing different classification and segmentation problems: *color*, *material*, and *material-color*. Table S9 and Fig. S12 in the Supplementary Material show the number of pixels labeled in each class and the mean spectral signatures available in the *HSI plastic dataset*. While VNIR data should accurately identify different colors (since this spectral range includes the visible range) and NIR data should identify more accurately different materials^39^. The aim of dividing the data into these groups (*color*, *material*, and *material-color*) is to test the hypothesis that the fusion of both sources of information may offer the best discrimination in a classification problem where materials and colors should be differentiated simultaneously. All datasets are described in detail in the “Methods” section in the Supplementary Material and are available under reasonable request.

### Data pre-processing

The raw HS images acquired by both cameras were pre-processed applying image calibration to avoid the influence of environmental illumination, the dark currents of the HS sensor, and noise filtering to reduce the high-frequency noise in the spectral signatures caused by the camera sensor. Additionally, due to both HS cameras have different spatial resolutions, it was necessary to resample one of the two HS images to be able to register them. Upsampling method was chosen to increase the NIR spatial dimensions to reach the VNIR pixel size. Then the VNIR image is employed to perform a manual labeling and such labeling map was transferred to the upampled NIR image. The upsampling algorithm used to increase the NIR spatial resolution (from 320 × 253 to 939 × 743 pixels) and to estimate the upsampled spectral signatures is based on a bilinear interpolation, considering the nearest 2-by-2 neighborhood of a certain pixel. Figure S13 in the Supplementary Material shows a graphical representation of this methodology. In the “Methods” section in the Supplementary Material, data pre-processing and upsampling methods are detailed and a comparison between three different interpolation methods is provided.

### VNIR–NIR spatial registration

In this study, intensity-based, using translation, similarity, and affine transformation, and feature-based techniques, using SURF^40^ and MSER^41^ detectors and similarity, affine and projective transformation, were employed for registering the VNIR and NIR images. This process allows overlapping two or more images of the same scene captured by different cameras and different angles using a reference image. In this work, the misaligned image (also called *moving image*) was the NIR image, and the reference image (also called *fixed image*) corresponded to the VNIR image. Finally, after applying the transformation to the NIR image, both VNIR and NIR images were cropped to obtain the same region of interest. In the “Methods” section in the Supplementary Material, the techniques and transformation used are explained.

### VNIR–NIR spectral fusion

The final step of the proposed framework aims to combine the spectra from the registered NIR and VNIR HS images into a single HS image. First, a spectral analysis of the data generated in both HS images was performed to evaluate the optimal spectral cutoff points where the HS sensors present low performance, i.e., low signal-to-noise ratios. The lower and higher spectral bands were removed before the data fusion. The spectral range of 400–435 nm and 901–1000 nm of the VNIR and the range of 900–956 nm and 1638–1700 nm of the NIR were not included in the fused data as explained in the “Results” section. Then, a reflectance offset was applied to NIR spectrum with the goal of adjusting the reflectance values of both spectral signatures with respect to a reference. The reflectance offset is detailed in the “Methods” section in the Supplementary Material.

Thus, the fused HS image was formed by the spectral ranges of 435–901 nm (641 spectral bands) and 956–1638 nm (144 spectral bands) as shown in Fig. 5, where the spectral signature of Zenith Polymer Reflectance Standard provided by the manufacturer is used to represent the spectral range after the fusion process. Figure 2f shows the VNIR–NIR spectral fusion result using such polymer acquired with the HS acquisition system. Finally, the spectral signatures are normalized between zero and one to homogenize reflectance levels in each pixel of the HS image for the subsequent segmentation and classification analyses. Figure S12 in Supplementary Material shows the average spectral signatures of the HSI plastic dataset after performing the VNIR–NIR spectral fusion.

Additionally, the fusion performance was evaluated using segmentation and classification algorithms, comparing the results before and after the proposed fusion procedure using the *HSI plastic dataset*. The obtained results are detailed in the *VNIR*–*NIR Spectral Fusion Methods Evaluation* section in “Results” section of the Supplementary Material.

### Segmentation and classification methods

The VNIR–NIR imaging fusion performance was evaluated in unsupervised segmentation and supervised classification problems. The goal was to quantitatively and qualitatively determine if the proposed fusion approach allows to improve the segmentation and classification of different classes with respect to the exclusive use of either VNIR or NIR data.

The segmentation method employed the K-means, K-medoids, and hierarchical K-means algorithms^36^ to segment the HS images into *K* different clusters. The number of clusters (*K*) was previously selected and, in the case of HSI plastic dataset, the selected *K* value corresponds to the number of classes present in the ground-truth of each HS image to be processed. In the case of HSI brain dataset, the number of clusters used was twenty-four. This number was selected based on the results of a previous work^36^. Finally, to obtain the segmentation maps, the clusters more similar to the ground-truth were selected using Jaccard metric. In these experiments, the clusters initialization was performed using the same seed. K-means and hierarchical K-means algorithms have been used for HS data segmentation to identify brain cancer^36,42^. MATLAB Statistics and Machine Learning Toolbox (The MathWorks Inc., Natick, MA, USA) was employed to implement the K-means algorithms.

The pixel-wise supervised classification was based on the SVM, RF, KNN classifiers. In the classification problem, the *HSI plastic dataset* was partitioned into training, validation, and test sets. The training and validation sets were used to optimize, evaluate, and generate the classification model. After the hyperparameter optimization, the performance of the model was evaluated using the test set. These algorithm has been widely used to identify glioblastoma tumor in pathological slide and in-vivo tissue using HS data^43,44^. The LIBSVM library was used as SVM implementation^45^ while the MATLAB Statistics and Machine Learning ToolBox was employed for the RF and KNN implementations. More details can be found in the “Methods” section in the Supplementary Material.

### Performance metrics

The spatial registration was evaluated using image-based similarity, while the segmentation problem performance was evaluated using overlap-based metrics. Finally, the classification problem was evaluated using the accuracy metric Additionally, segmentation and classification results were statistically analyzed using a paired, one-tailed Student’s *t* test at the 5% significance level. Each evaluation metrics used in this research is detailed in the “Methods” section in the Supplementary Material.

## Data availability statement

The datasets generated during the current study are available from the corresponding author, under reasonable request, through https://hsibraindatabase.iuma.ulpgc.es/.

## Supplementary Information

Supplementary Information.
