# Supplementary material for: VNIR–NIR hyperspectral imaging fusion targeting intraoperative brain cancer detection
Source: Sci Rep. 2021 Oct 4;11:19696. doi: 10.1038/s41598-021-99220-0 (PMC8490425; doi:10.1038/s41598-021-99220-0)
Supplement: Supplementary file 1 — Supplementary Information. [file 41598_2021_99220_MOESM1_ESM.pdf]

VNIR-NIR Hyperspectral Imaging Fusion Targeting Intraoperative Brain Cancer Detection

Raquel Leon[1,*,+], Himar Fabelo[1,*,+], Samuel Ortega[1,+], Juan F. Piñeiro[2], Adam Szolna[2], Maria Hernandez[2], Carlos Espino[2], Aruma J. O'Shanahan[2], David Carrera[2], Sara Bisshopp[2], Coralia Sosa[2], Mariano Marquez[2], Jesus Morera[2], Bernardino Clavo[3], and Gustavo M. Callico[1,*]

[1]Institute for Applied Microelectronics, University of Las Palmas de Gran Canaria, Las Palmas de Gran Canaria, 35017, Spain.
[2]Department of Neurosurgery, University Hospital Doctor Negrin of Gran Canaria, Barranco de la Ballena s/n, Las Palmas de Gran Canaria, 35010, Spain.
[3]Research Unit, University Hospital Doctor Negrin of Gran Canaria, Instituto de Investigación Sanitaria de Canarias (IISC), Barranco de la Ballena s/n, Las Palmas de Gran Canaria, 35010, Spain.
[*]slmartin@iuma.ulpgc.es; hfabelo@iuma.ulpgc.es; gustavo@iuma.ulpgc.es;
[+]these authors contributed equally to this work.

Supplementary Figures

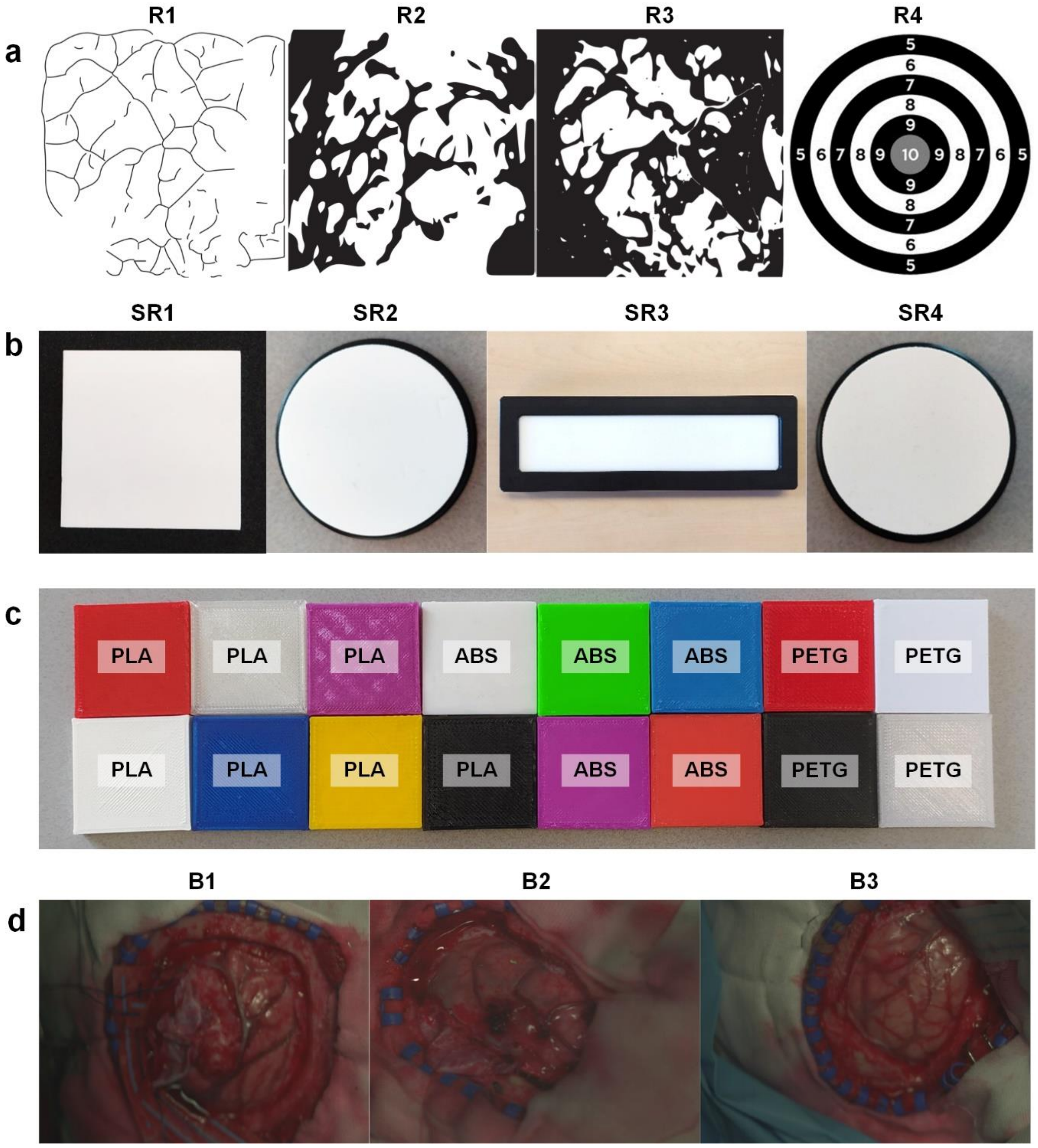

Figure S1. HS datasets used in this research. (a) Patterns based on brain morphological structures and a shooting target employed to generate the *HSI registration dataset* (seven HS images) used to evaluate the image registration techniques. (b) Three different 99% Spectralon White Diffuse Reflectance Standards and a Zenith Polymer Reflectance Standard used to obtain the *HSI spectral reference dataset* (seven HS images) employed to evaluate the proposed spectral fusion approach. (c) Sixteen square plastic samples of different colors and three materials: polylactic acid (PLA), acrylonitrile butadiene styrene (ABS), and polyethylene terephthalate glycol (PETG). These samples were employed to generate the *HSI plastic dataset* (twenty HS images), which was used to qualitatively and quantitatively evaluate the fusion performance. (d) Pseudo-RGB images of the *HSI brain dataset* (three HS images) utilized to qualitatively evaluate the fusion performance.

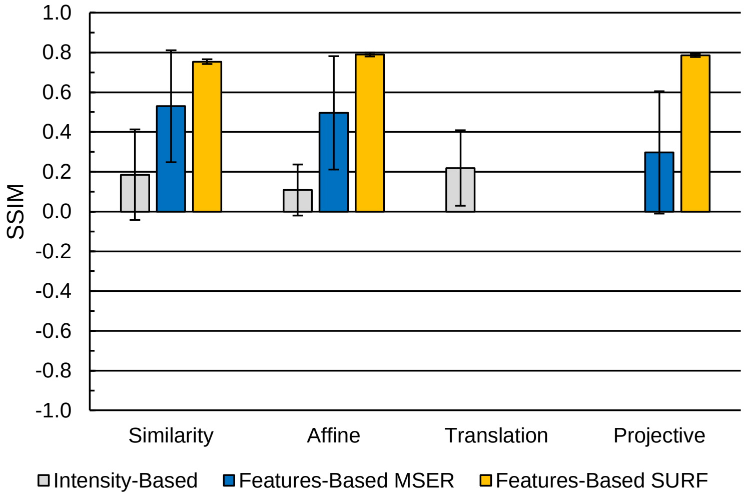

(a)

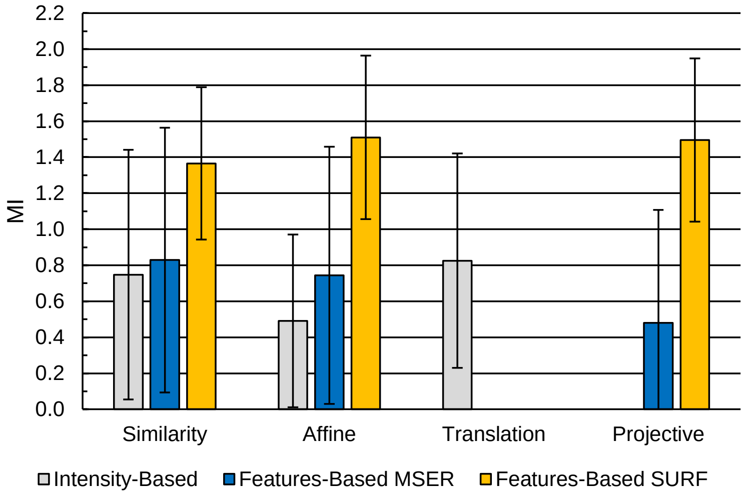

(b)

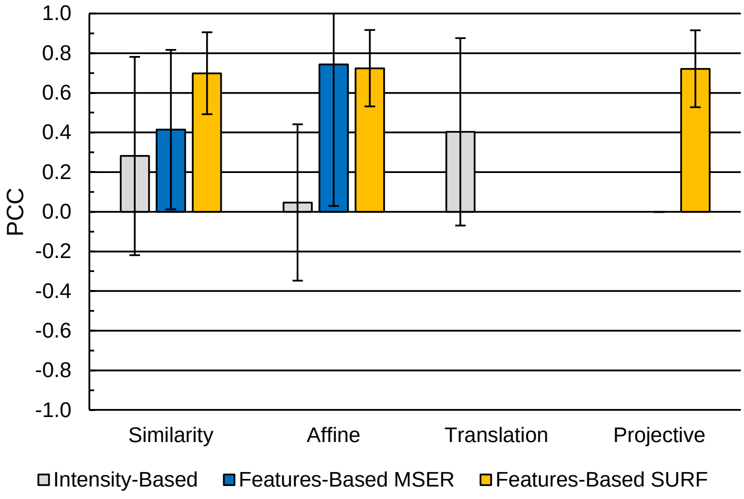

(c)

Figure S2. Average image registration results using the gray-scale representation of the pseudo-RGB images from *HSI registration dataset* applying intensity-based techniques with similarity, affine, and translation transformations and featured-based using Maximally Stable Extremal Regions (MSER) and Speeded Up Robust Features (SURF) detector and similarity, affine and projective transformation. (a, b, c) Structural Similarity Index Measure (SSIM), Mutual Information (MI), Pearson's Correlation Coefficient (PCC) values, respectively.

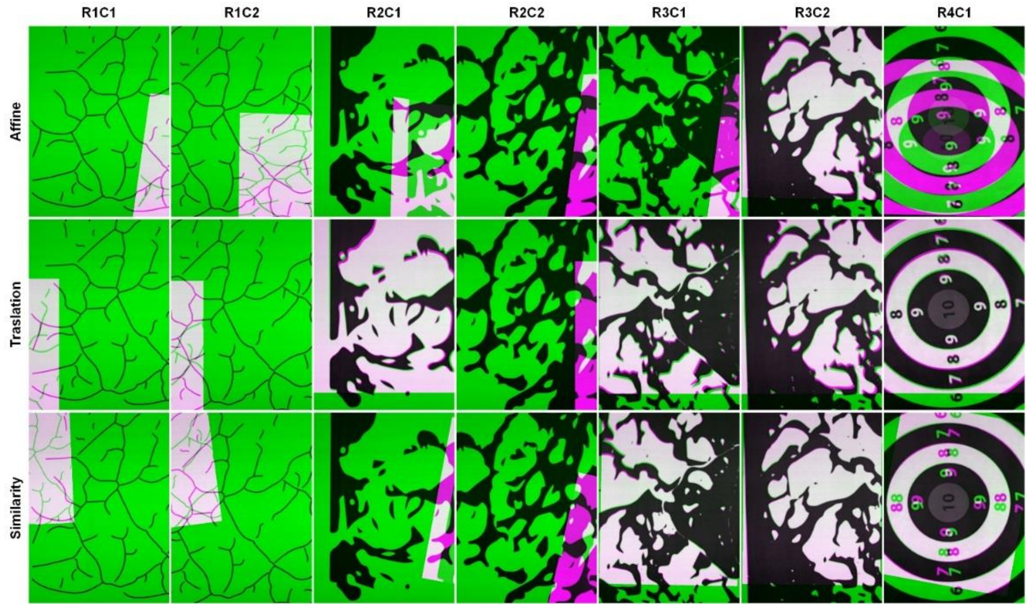

(a)

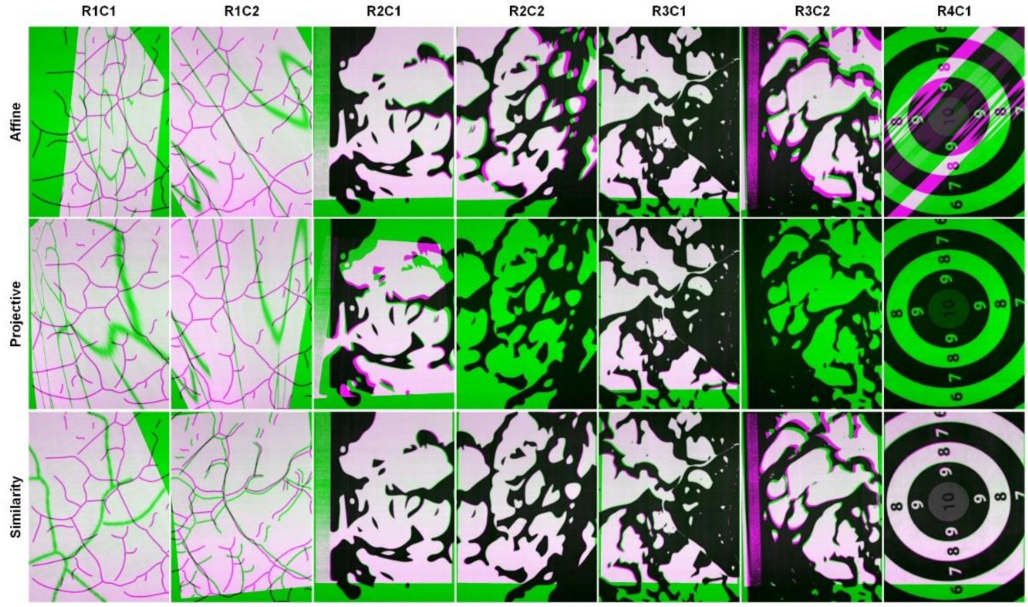

(b)

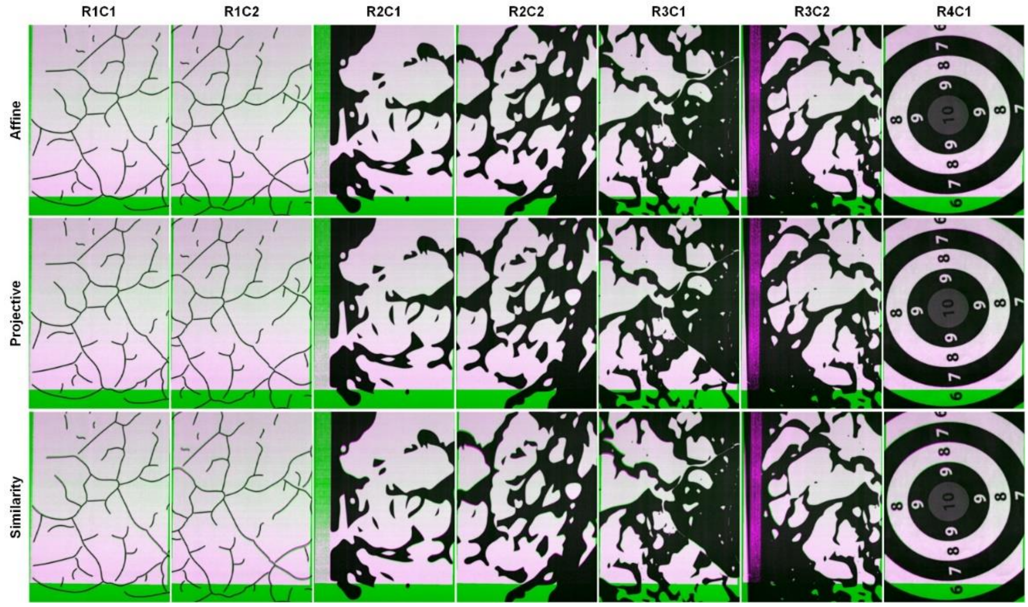

(c)

Figure S3. VNIR-NIR Spatial Registration using the *HSI registration dataset*. Both images are overlapped using green-magenta false-color, VNIR (green) and NIR (magenta). (a) Intensity-based technique. (b) Feature-based technique using MSER detector. (c) Feature-based technique using SURF detector.

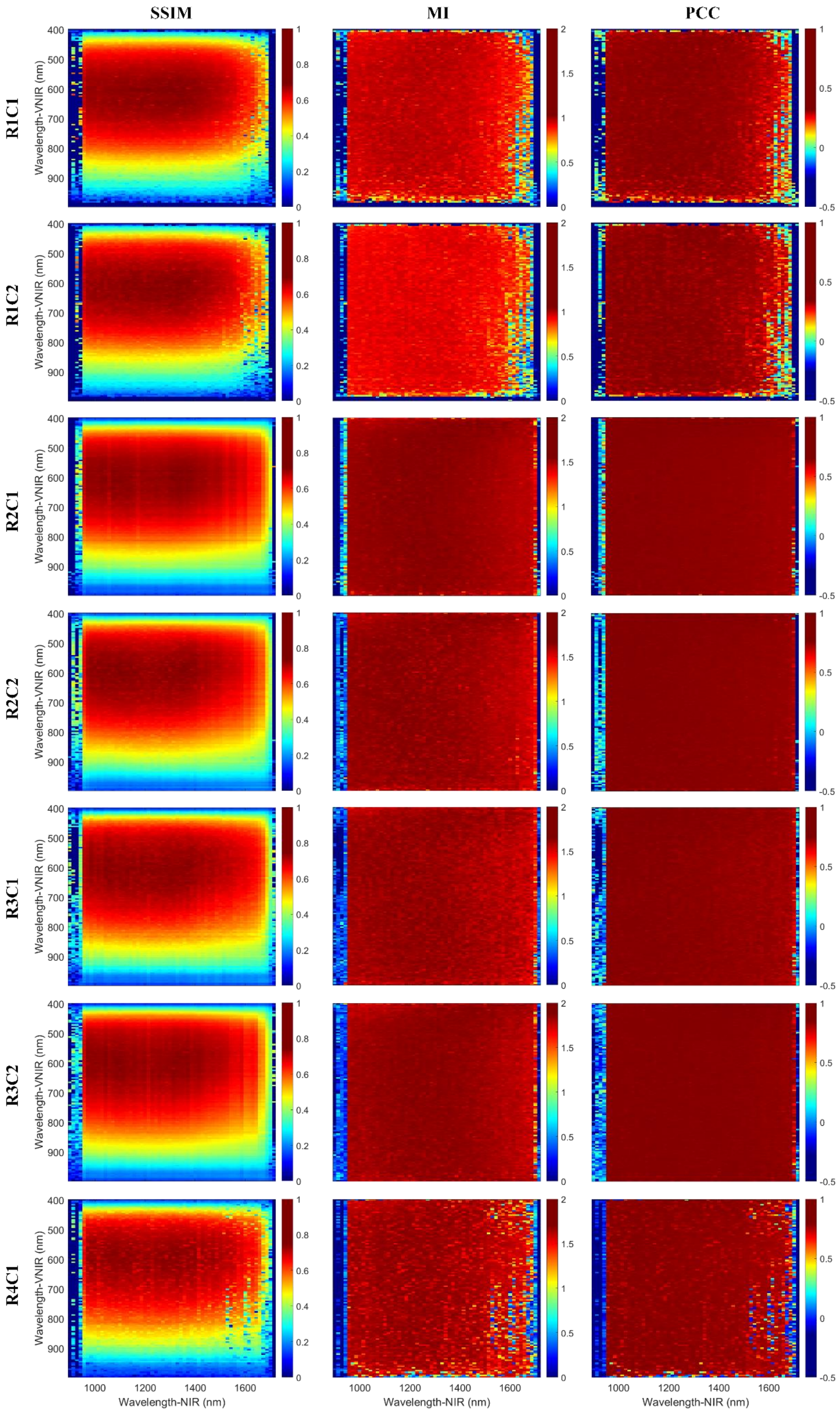

Figure S4. Coarse search results of Structural Similarity Index Measure (SSIM), Mutual Information (MI), and Pearson's Correlation Coefficient (PCC) for identifying the suitable spectral bands for the registration applying the feature-based SURF technique with projective transformation to the *HSI registration dataset*.

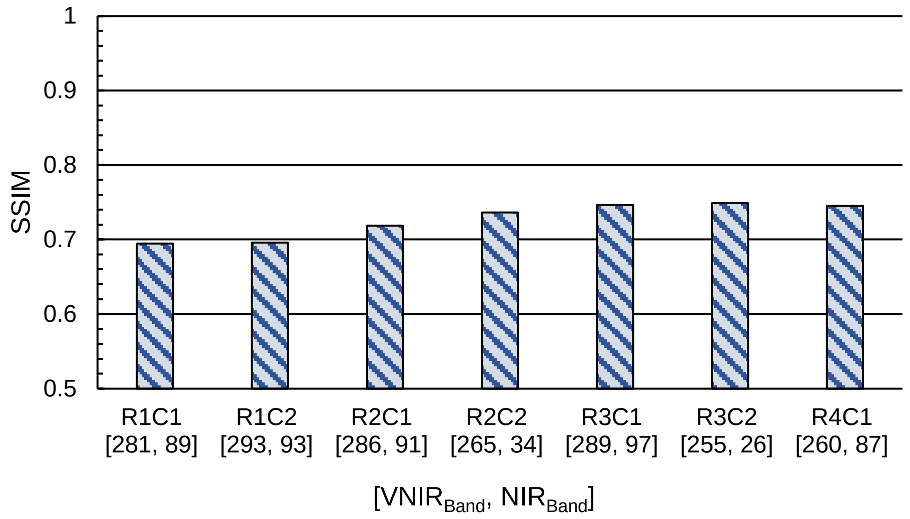

Figure S5. Structural Similarity Index Measure (SSIM) value using the best spectral band for each HS image from the *HSI registration dataset*. The relation between the band number and the wavelength in the VNIR is the following: $281 = 604.20\ nm$; $293 = 612.93\ nm$; $286 = 607.84\ nm$; $265 = 592.56\ nm$; $289 = 610.02\ nm$; $255 = 585.28\ nm$; $260 = 588.92\ nm$; and in the NIR is the following: $89 = 1318.95\ nm$; $93 = 1338.02\ nm$; $91 = 1328.48\ nm$; $34 = 1056.76\ nm$; $97 = 1357.09\ nm$; $26 = 1018.62\ nm$; $87 = 1309.42\ nm$.

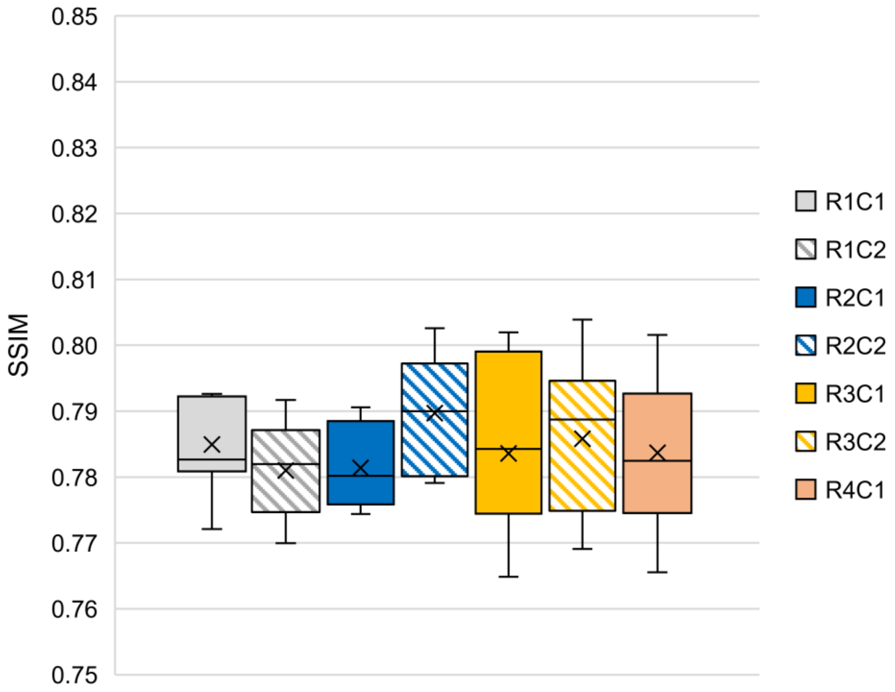

Figure S6. Structural Similarity Index Measure (SSIM) results using the seven different transformation models obtained using the optimal spectral bands for each image pair. The box boundaries represent the IQR (Interquartile Range) of the results. Central bars and error bars depict median and minimum/maximum values of SSIM, respectively.

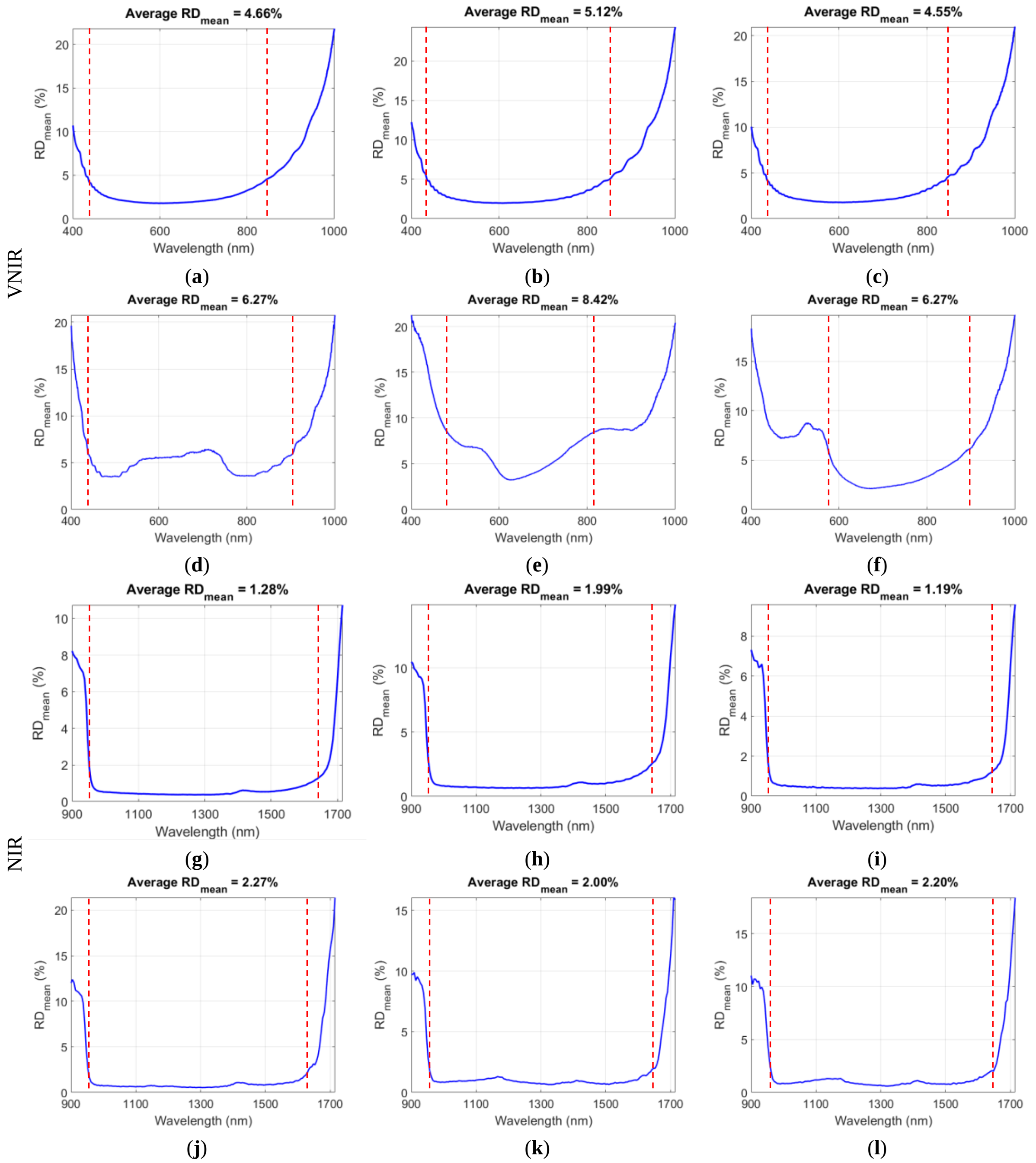

Figure S7. Absolute relative difference percentage (RD) results using the three images from the *HSI spectral reference dataset* and three images from *HSI plastic dataset*. (a-f) RD results using VNIR images. (g-l) RD results using NIR images.

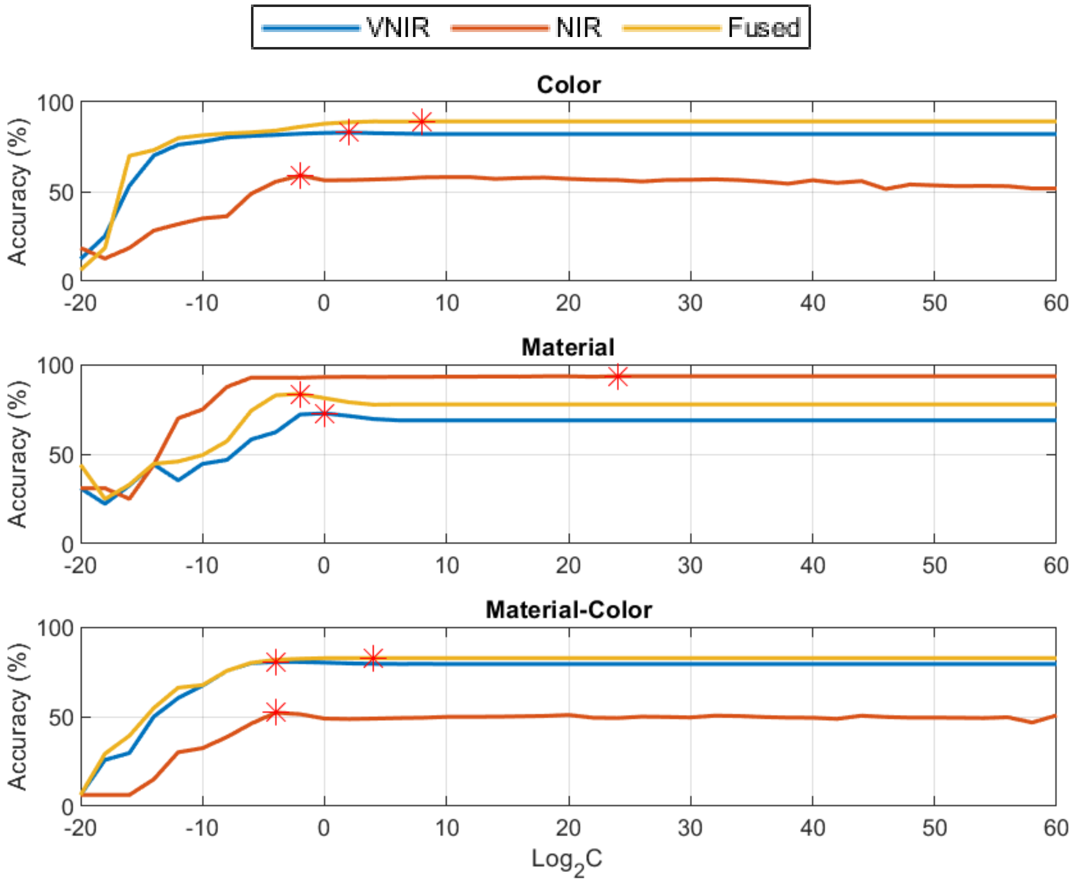

Figure S8. Coarse search representation of the accuracy results obtained for SVM Linear classifier modifying the cost (*C*) hyperparameter in each classifier problem (*color*, *material*, and *material-color*) and each HS data type (VNIR, NIR, and Fused).

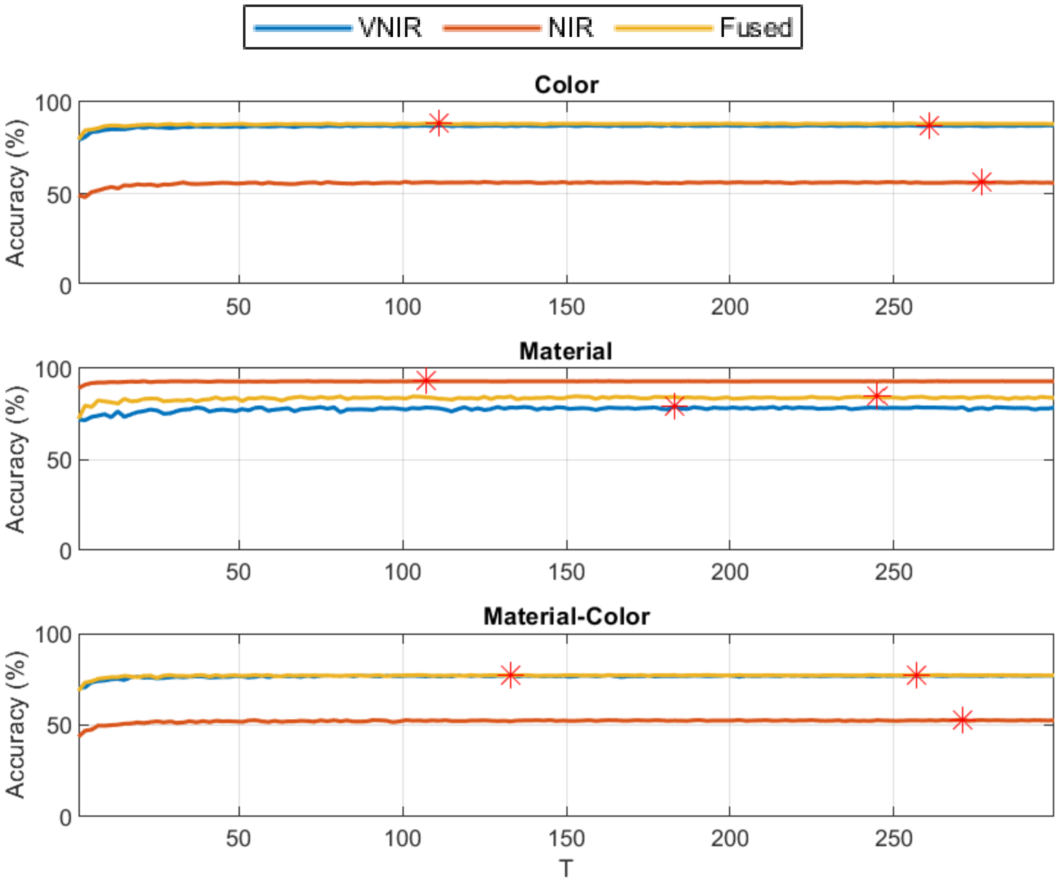

Figure S9. Coarse search representation of the accuracy results obtained for RF classifier modifying the number of trees (T) hyperparameter in each classifier problem (*color*, *material*, and *material-color*) and each HS data type (VNIR, NIR, and Fused).

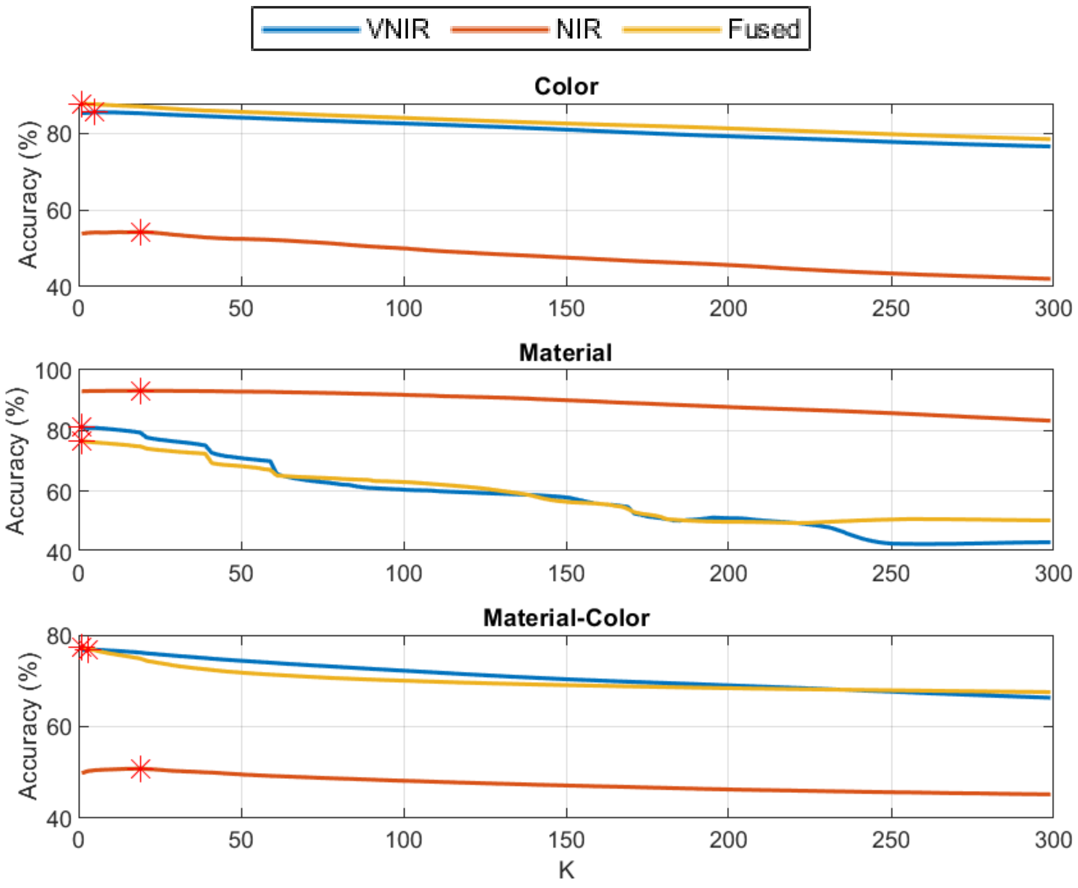

Figure S10. Coarse search representation of the accuracy results obtained for KNN classifier modifying the number of nearest neighbors (K) hyperparameter in each classifier problem (*color*, *material*, and *material-color*) and each HS data type (VNIR, NIR, and Fused).

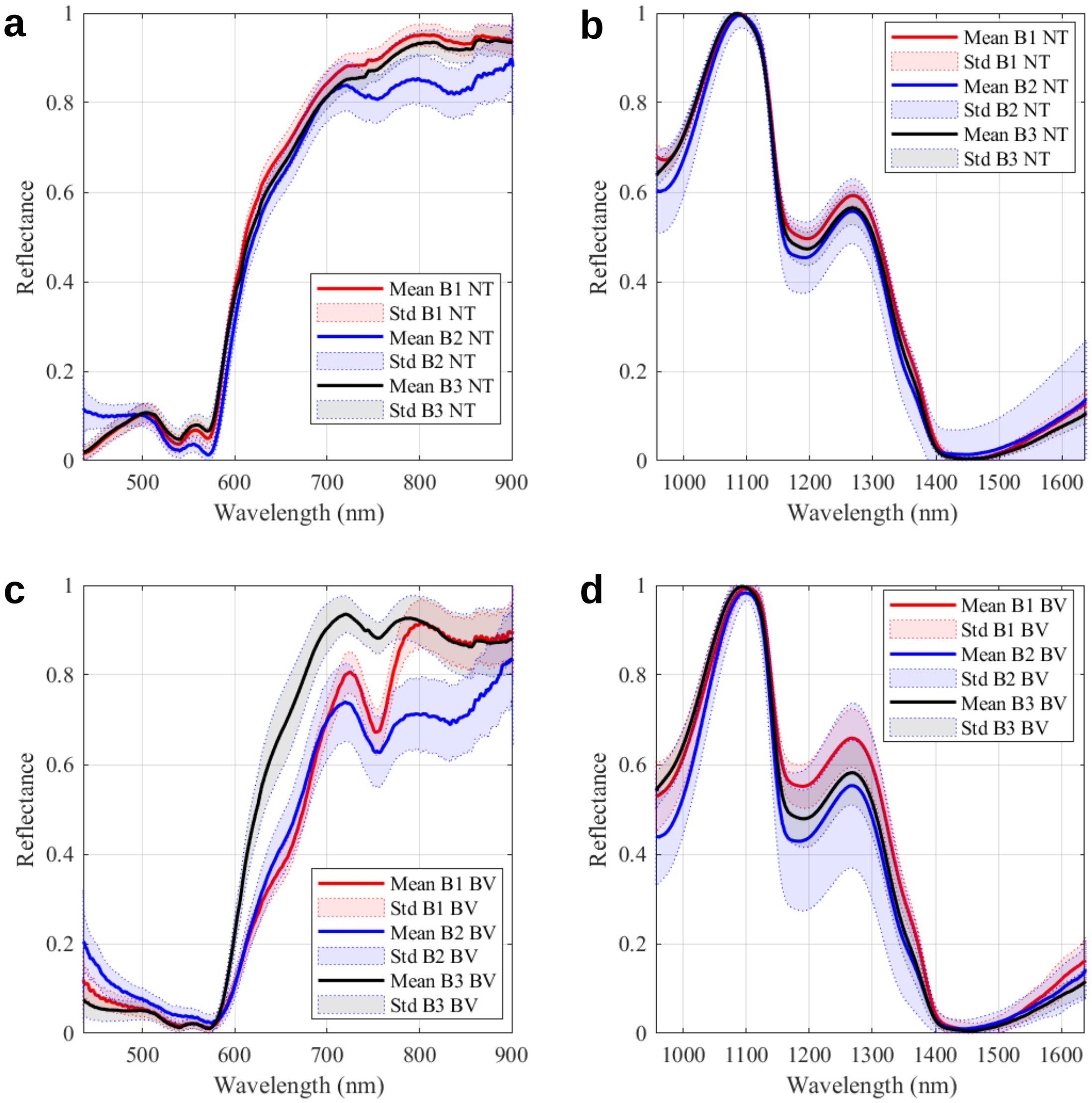

Figure S11. Average and standard deviations of the labeled spectral signatures of the *HSI brain dataset*. (**a, b**) Spectral signatures of the normal tissue pixels in the VNIR and NIR regions, respectively. (**c**, **d**) Spectral signatures of the blood vessels in the VNIR and NIR regions, respectively. NT: Normal Tissue; BV: Blood Vessels. Red, blue, and black colors represent the B1, B2, and B3 HS images from the *HSI brain dataset* respectively.

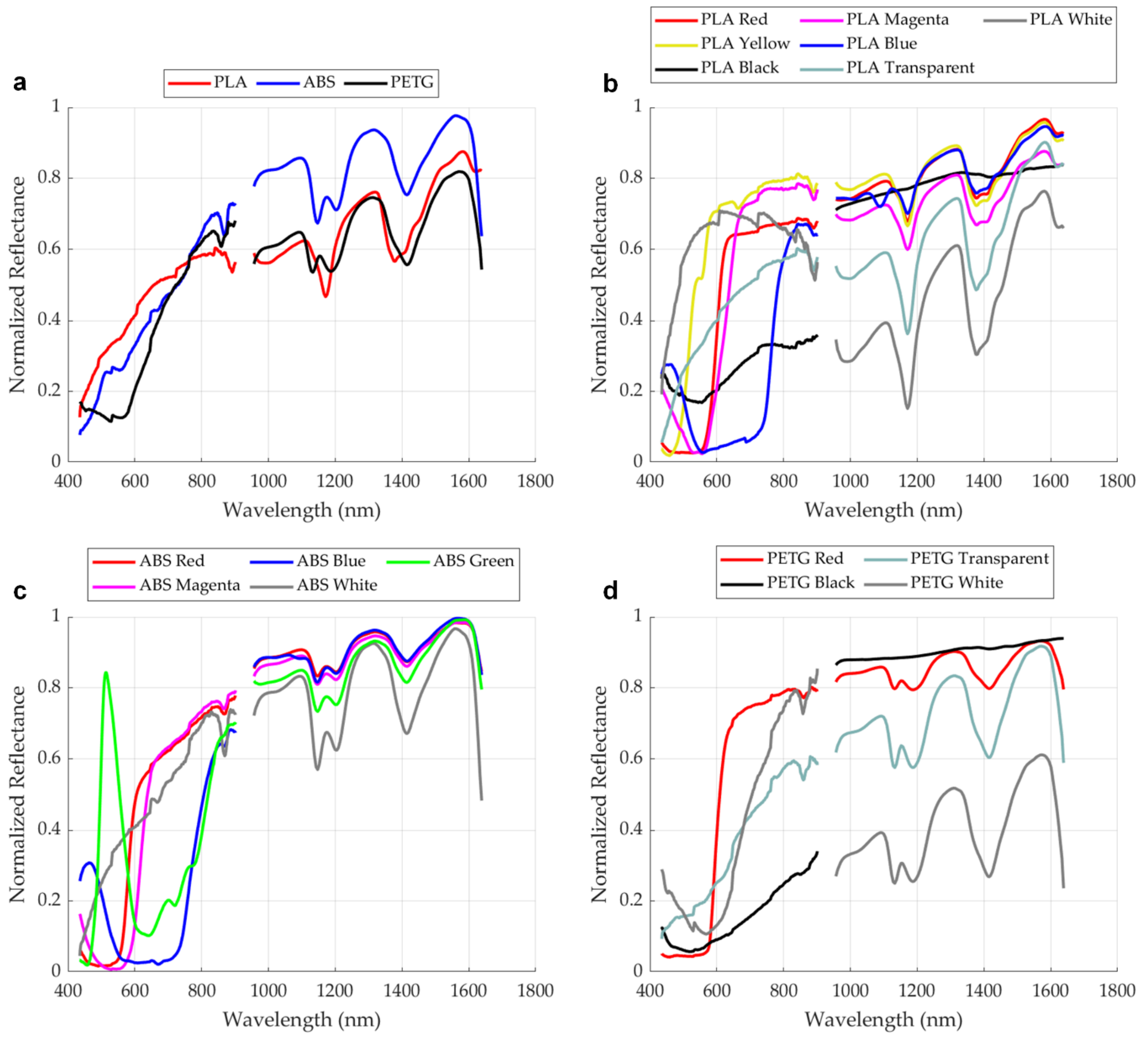

Figure S12. Average spectral signatures of the Fused data from the *HSI plastic dataset*. (**a**) Material spectral signatures. (**b**, **c**, **d**) Color spectral signatures from polylactic acid (PLA), acrylonitrile butadiene styrene (ABS), and polyethylene terephthalate glycol (PETG) materials.

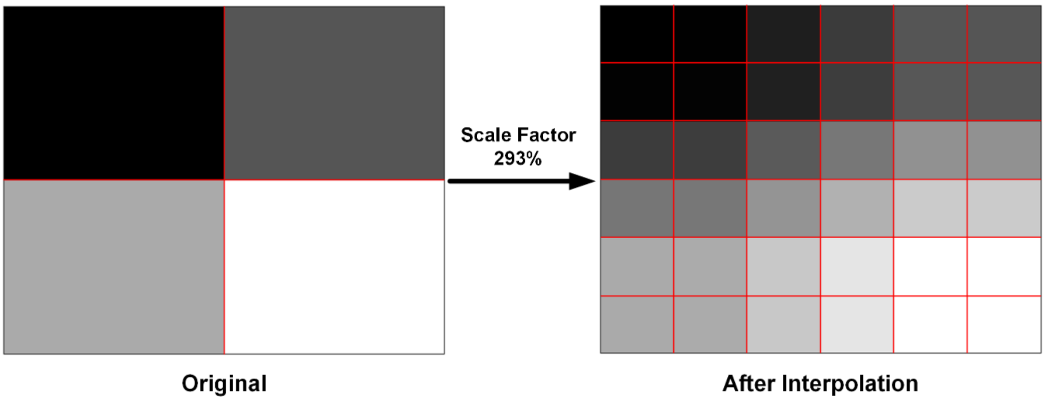

Figure S13. Graphical representation of NIR spatial upsampling using bilinear interpolation with a scale factor of 293%. Original image size: 320 × 253 pixels before interpolation and 939 × 743 pixels after interpolation.

Supplementary Tables

		Initial Cutoff Point		Final Cutoff Point	
		Wavelength (nm)	#Band	Wavelength (nm)	#Band
**VNIR**	**SR1**	435.40	49	849.36	618
	**SR2**	434.67	48	846.48	614
	**SR3**	435.40	49	853.03	623
	**Blue ABS**	438.31	53	906.87	697
	**Red PLA**	476.87	106	879.22	659
	**Magenta PLA**	574.37	240	901.78	689
**NIR**	**SR1**	956.64	13	1638.35	156
	**SR2**	956.64	13	1638.35	156
	**SR3**	956.64	13	1619.28	152
	**Blue ABS**	956.64	13	1628.82	154
	**Red PLA**	961.41	14	1638.35	156
	**Magenta PLA**	961.41	14	1647.88	158

Table S1. Spectral analysis using *HSI spectral reference dataset*. The wavelength and the band numbers of the initial and final cutoff point were computed using absolute relative difference percentage for VNIR and NIR images.

	Color			Material			Material-Color		
	**VNIR**	**NIR**	**Fused**	**VNIR**	**NIR**	**Fused**	**VNIR**	**NIR**	**Fused**
**R1**	**0.97**	0.95	0.77	0.52	**0.79**	0.41	0.21	**0.51**	0.41
**R2**	0.77	0.63	**0.87**	**0.56**	0.24	0.32	**0.56**	0.24	0.42
**R3**	0.55	**0.92**	0.52	0.67	**0.88**	0.34	**0.67**	0.44	0.51
**R4**	0.39	**0.92**	0.51	0.27	**0.93**	0.31	0.27	**0.93**	0.31
**R5**	**0.96**	0.89	0.94	0.36	**0.91**	0.73	0.49	**0.91**	0.73
**R6**	**0.94**	**0.94**	0.69	0.86	**0.89**	0.48	0.40	**0.89**	0.48
**R7**	**0.84**	0.37	0.63	0.64	**0.93**	0.55	0.52	0.29	**0.63**
**R8**	**0.93**	0.45	0.48	0.39	0.45	**0.63**	**0.93**	0.45	0.90
**R9**	**0.71**	0.29	0.70	0.39	**0.93**	0.56	0.55	0.38	**0.70**
**R10**	0.86	0.85	**0.87**	0.86	0.85	**0.87**	0.86	0.45	**0.87**
**R11**	**0.81**	0.70	0.52	0.81	0.70	**0.89**	0.81	0.48	**0.89**
**R12**	0.83	0.84	**0.87**	0.83	0.84	**0.87**	0.83	0.84	**0.87**
**R13**	**0.96**	0.65	0.94	0.43	**0.91**	0.42	**0.85**	0.63	0.70
**Avg.**	**0.81**	0.72	0.72	0.58	**0.79**	0.57	0.61	0.57	**0.65**
**Std.**	**0.17**	0.23	**0.17**	**0.21**	**0.21**	**0.21**	0.23	0.24	**0.20**

Table S2. Jaccard results of the K-means segmentation of each HS image employing the test set from the *HSI plastic dataset* and their average (avg) and standard deviation (std). The results are divided into *color*, *material*, and *material-color* segmentation problems, using VNIR, NIR, and fused data.

	Color			Material			Material-Color		
	**VNIR**	**NIR**	**Fused**	**VNIR**	**NIR**	**Fused**	**VNIR**	**NIR**	**Fused**
**R1**	**0.97**	0.95	0.85	0.31	**0.80**	0.41	0.28	**0.80**	0.41
**R2**	0.24	0.64	**0.87**	**0.55**	0.51	0.42	**0.56**	0.37	0.42
**R3**	0.39	**0.92**	0.55	0.67	**0.88**	0.60	**0.67**	0.43	0.60
**R4**	0.40	**0.91**	0.49	0.27	**0.92**	0.29	0.28	**0.92**	0.29
**R5**	**0.96**	0.88	0.94	0.83	**0.91**	0.74	0.33	**0.91**	0.74
**R6**	**0.94**	0.93	0.74	0.88	**0.90**	0.52	0.40	**0.91**	0.43
**R7**	**0.85**	0.30	0.73	0.64	**0.93**	0.57	**0.85**	0.29	0.73
**R8**	0.49	**0.57**	0.49	0.66	**0.97**	0.65	**0.92**	0.57	0.49
**R9**	0.58	0.39	**0.70**	0.40	**0.93**	0.39	**0.71**	0.29	0.70
**R10**	0.85	0.45	**0.86**	0.85	0.85	**0.86**	0.85	0.85	**0.86**
**R11**	0.80	0.47	**0.89**	0.80	0.75	**0.87**	**0.80**	0.75	0.43
**R12**	0.83	0.84	**0.87**	0.83	0.46	**0.87**	0.83	0.84	**0.86**
**R13**	**0.96**	0.65	0.94	0.43	**0.66**	0.42	0.60	0.63	**0.72**
**Avg.**	0.71	0.68	**0.76**	0.63	**0.81**	0.58	0.62	**0.66**	0.59
**Std.**	0.26	0.24	**0.16**	0.22	**0.17**	0.20	0.23	0.24	**0.19**

Table S3. Jaccard results of the K-medoids segmentation of each HS image employing the test set from the *HSI plastic dataset* and their average (avg) and standard deviation (std). The results are divided into *color*, *material*, and *material-color* segmentation problems, using VNIR, NIR, and fused data.

	**Color**			**Material**			**Material-Color**		
	**VNIR**	**NIR**	**Fused**	**VNIR**	**NIR**	**Fused**	**VNIR**	**NIR**	**Fused**
**R1**	**0.97**	0.95	0.77	0.52	**0.76**	0.33	0.52	**0.76**	0.33
**R2**	0.24	0.63	**0.87**	**0.55**	0.30	0.41	**0.55**	0.30	0.41
**R3**	0.55	**0.92**	0.52	0.67	**0.89**	0.31	0.67	**0.89**	0.34
**R4**	0.39	**0.92**	0.51	0.23	**0.93**	0.29	0.19	**0.93**	0.29
**R5**	**0.96**	0.89	0.94	0.49	**0.91**	0.73	0.49	**0.91**	0.73
**R6**	**0.94**	**0.94**	0.69	0.40	**0.89**	0.48	0.40	**0.89**	0.48
**R7**	**0.81**	0.38	0.62	0.36	**0.93**	0.55	**0.81**	0.38	0.62
**R8**	**0.91**	0.57	**0.91**	0.39	**0.96**	0.40	**0.91**	0.57	**0.91**
**R9**	0.63	0.40	**0.64**	0.39	**0.93**	0.56	0.63	0.40	**0.64**
**R10**	0.82	**0.85**	0.71	0.82	**0.85**	0.71	0.82	**0.85**	0.71
**R11**	0.80	0.78	**0.89**	0.80	0.78	**0.89**	0.80	0.78	**0.89**
**R12**	0.81	0.74	**0.85**	0.81	0.74	**0.85**	0.81	0.74	**0.85**
**R13**	**0.95**	0.57	0.93	0.42	**0.59**	0.41	**0.85**	0.60	0.71
**Avg.**	0.75	0.73	**0.76**	0.53	**0.80**	0.53	0.65	**0.69**	0.61
**Std.**	0.23	0.21	**0.15**	0.19	**0.18**	0.20	**0.21**	0.22	0.22

Table S4. Jaccard results of the Hierarchical K-means segmentation of each HS image employing the test set from the *HSI plastic dataset* and their average (avg) and standard deviation (std). The results are divided into *color*, *material*, and *material-color* segmentation problems, using VNIR, NIR, and fused data.

Classifier	HP	Sensor	Method	Coarse Search		Fine Search		Accuracy (%)
				I/S/F	Optimal	I/S/F	Optimal	
SVM-Linear	C	VNIR	Color	$2^{-20}/2^{2}/2^{60}$	2^{2}	$2^{0}/2^{0.5}/2^{4}$	2^{2}	82.93
			Material	$2^{-20}/2^{2}/2^{60}$	2^{0}	$2^{-2}/2^{0.5}/2^{2}$	$2^{0.5}$	72.83
			Material-Color	$2^{-20}/2^{2}/2^{60}$	2^{-4}	$2^{2}/2^{0.5}/2^{6}$	2^{2}	79.68
		NIR	Color	$2^{-20}/2^{2}/2^{60}$	2^{-2}	$2^{-4}/2^{0.5}/2^{0}$	$2^{-2.5}$	59.30
			Material	$2^{-20}/2^{2}/2^{60}$	2^{2}	$2^{-2}/2^{0.5}/2^{2}$	2^{1}	**93.10**
			Material-Color	$2^{-20}/2^{2}/2^{60}$	2^{-4}	$2^{-6}/2^{0.5}/2^{-2}$	2^{-3}	53.25
		Fusion	Color	$2^{-20}/2^{2}/2^{60}$	2^{8}	$2^{6}/2^{0.5}/2^{10}$	2^{7}	**89.15**
			Material	$2^{-20}/2^{2}/2^{60}$	2^{-2}	$2^{-4}/2^{0.5}/2^{0}$	2^{-3}	83.56
			Material-Color	$2^{-20}/2^{2}/2^{60}$	2^{4}	$2^{2}/2^{0.5}/2^{6}$	2^{5}	**82.65**
RF	T	VNIR	Color	1/2/300	261	-	-	87.33
			Material	1/2/300	183	-	-	79.17
			Material-Color	1/2/300	133	-	-	77.37
		NIR	Color	1/2/300	277	-	-	56.47
			Material	1/2/300	107	-	-	**93.01**
			Material-Color	1/2/300	271	-	-	52.98
		Fusion	Color	1/2/300	111	-	-	**88.39**
			Material	1/2/300	245	-	-	84.65
			Material-Color	1/2/300	257	-	-	**77.43**
KNN	K	VNIR	Color	1/2/300	5	-	-	85.47
			Material	1/2/300	1	-	-	81.03
			Material-Color	1/2/300	3	-	-	**76.82**
		NIR	Color	1/2/300	19	-	-	54.24
			Material	1/2/300	19	-	-	**93.16**
			Material-Color	1/2/300	19	-	-	50.86
		Fusion	Color	1/2/300	1	-	-	**87.47**
			Material	1/2/300	1	-	-	76.50
			Material-Color	1/2/300	1	-	-	77.38

Table S5. Coarse-to-fine search to optimize the Cost (C), the number of trees (T) and number of nearest neighbors (K) hyperparameters of the SVM, RF, and KNN classifiers using the validation set of the *HSI plastic dataset*. Fine search was not performed in RF and KNN algorithm because the execution time in these classifiers is lower than SVM classifier. HP: Hyperparameter; I: Initial value; S: Step value; F: Final value

	Color			Material			Material-Color		
	**VNIR**	**NIR**	**Fused**	**VNIR**	**NIR**	**Fused**	**VNIR**	**NIR**	**Fused**
**R1 (%)**	**97.0**	52.0	**97.0**	93.4	94.8	**97.4**	**94.2**	69.9	**94.2**
**R2 (%)**	86.1	**96.4**	82.2	48.2	**61.6**	47.9	45.9	**52.4**	44.9
**R3 (%)**	**77.3**	59.2	72.0	75.7	**96.0**	95.4	69.8	56.0	**72.1**
**R4 (%)**	73.8	61.8	**76.9**	59.2	**96.4**	92.8	59.0	51.9	**95.1**
**R5 (%)**	**97.6**	97.1	93.9	85.4	**97.7**	93.3	93.2	**96.4**	93.1
**R6 (%)**	**97.4**	43.5	96.1	95.7	**96.9**	96.0	**95.6**	33.0	94.9
**R7 (%)**	**82.1**	59.4	77.2	97.4	97.2	**98.2**	**81.7**	52.3	74.4
**R8 (%)**	95.7	63.2	**96.6**	82.4	**97.7**	96.9	76.1	67.4	**91.9**
**R9 (%)**	**93.0**	64.7	92.8	95.8	**97.1**	**97.1**	91.1	65.7	**94.9**
**R10 (%)**	93.2	70.0	**96.4**	71.8	**89.8**	85.0	69.6	79.1	**85.2**
**R11 (%)**	88.4	70.1	**92.3**	83.6	86.6	**90.5**	76.1	43.7	**81.8**
**R12 (%)**	94.0	80.8	**96.5**	70.5	87.2	**88.4**	59.4	78.7	**80.9**
**R13 (%)**	**97.7**	71.1	97.4	86.5	**95.6**	90.3	95.0	73.8	**96.5**
**Avg. (%)**	**90.2**	68.4	89.8	80.4	**91.9**	89.9	77.4	63.1	**84.6**
**Std. (%)**	**8.1**	15.6	9.2	15.0	**9.9**	13.2	16.2	17.0	**14.5**

Table S6. Accuracy results for the SVM classification of each HS image employing the test set from the *HSI plastic dataset* and their average (avg) and standard deviation (std). The results are divided into *color*, *material*, and *material-color* segmentation problems, using VNIR, NIR, and Fused data.

	Color			Material			Material-Color		
	**VNIR**	**NIR**	**Fused**	**VNIR**	**NIR**	**Fused**	**VNIR**	**NIR**	**Fused**
**R1 (%)**	97.5	61.3	**97.6**	85.7	**95.1**	94.1	94.2	64.4	**96.2**
**R2 (%)**	93.8	**97.0**	74.1	45.7	**55.1**	47.4	45.1	**51.1**	39.5
**R3 (%)**	67.5	62.2	73.1	59.5	**95.7**	93.5	**73.7**	62.2	73.1
**R4 (%)**	72.5	62.6	**91.9**	62.6	**97.3**	86.3	64.1	61.8	**90.3**
**R5 (%)**	**96.6**	94.6	96.3	87.0	**97.0**	92.2	93.9	**94.1**	84.0
**R6 (%)**	**97.2**	39.0	96.8	62.0	**97.2**	93.9	91.5	36.7	**95.5**
**R7 (%)**	**85.0**	53.3	76.7	89.1	**94.7**	91.4	**79.1**	54.2	74.7
**R8 (%)**	**96.1**	52.7	94.4	47.7	**97.2**	94.6	74.3	61.5	**87.8**
**R9 (%)**	**93.6**	69.6	93.1	89.1	**96.6**	94.9	92.4	70.8	**93.6**
**R10 (%)**	93.1	71.7	**96.3**	73.8	86.6	**88.7**	74.3	69.2	**84.4**
**R11 (%)**	92.1	65.6	**94.3**	66.8	**88.2**	87.1	72.1	59.3	**87.7**
**R12 (%)**	93.9	68.3	**95.8**	65.8	**88.7**	74.2	63.4	66.3	**81.6**
**R13 (%)**	97.2	80.9	**97.4**	76.6	**96.3**	92.9	94.5	74.7	**95.0**
**Avg. (%)**	90.5	67.6	**90.6**	70.1	**91.2**	87.0	77.9	63.6	**83.4**
**Std. (%)**	9.7	16.2	**9.3**	14.9	**11.5**	13.1	15.1	**13.4**	15.1

Table S7. Accuracy results for the RF classification of each HS image employing the test set from the *HSI plastic dataset* and their average (avg) and standard deviation (std). The results are divided into *color*, *material*, and *material-color* segmentation problems, using VNIR, NIR, and Fused data.

	Color			Material			Material-Color		
	**VNIR**	**NIR**	**Fused**	**VNIR**	**NIR**	**Fused**	**VNIR**	**NIR**	**Fused**
**R1 (%)**	96.3	57.6	**97.0**	92.5	**98.0**	93.1	93.8	61.6	**94.5**
**R2 (%)**	87.9	**96.1**	64.2	44.0	**55.2**	40.1	42.1	**52.4**	35.8
**R3 (%)**	63.2	**69.8**	66.8	66.9	**96.3**	84.0	**71.5**	66.3	67.7
**R4 (%)**	77.7	56.8	**90.0**	60.1	**97.1**	77.5	59.7	57.5	**80.8**
**R5 (%)**	**97.3**	92.2	96.0	91.8	**97.6**	87.2	**94.7**	92.4	92.8
**R6 (%)**	**96.1**	36.7	95.5	91.5	**98.3**	76.7	**92.6**	34.9	85.4
**R7 (%)**	**76.7**	57.3	75.0	89.9	**97.6**	83.4	**72.6**	57.8	69.3
**R8 (%)**	**91.6**	60.1	90.5	77.0	**98.4**	77.1	75.5	61.4	**79.2**
**R9 (%)**	86.6	72.5	**91.3**	85.5	**97.0**	92.6	86.1	71.7	**90.6**
**R10 (%)**	78.6	73.4	**90.4**	63.3	87.8	78.1	64.1	71.9	**77.4**
**R11 (%)**	85.4	66.2	**92.8**	86.2	89.5	**91.3**	73.9	56.2	**88.0**
**R12 (%)**	79.2	72.2	**90.6**	59.7	**88.1**	69.0	59.0	67.6	**79.5**
**R13 (%)**	**97.5**	78.6	97.1	92.9	**96.9**	93.7	**95.4**	73.3	94.8
**Avg. (%)**	85.7	68.4	**87.5**	77.0	**92.1**	80.3	75.5	63.5	**79.7**
**Std. (%)**	**10.3**	15.7	11.3	16.3	**11.8**	14.3	16.6	**13.4**	15.9

Table S8. Accuracy results for the KNN classification of each HS image employing the test set from the *HSI plastic dataset* and their average (avg) and standard deviation (std). The results are divided into *color*, *material*, and *material-color* segmentation problems, using VNIR, NIR, and Fused data.

Problem	Class Name	#Original Pixels Training	#Reduced Pixels Training	#Pixels Validation	#Pixels Test
**Color**	Red	163,510	1,000	159,608	480,283
	Yellow	53,782	1,000	51,544	52,172
	Black	113,084	1,000	109,323	280,492
	Magenta	108,124	1,000	106,648	216,632
	Blue	111,145	1,000	109,319	219,221
	Transparent	110,026	1,000	109,660	167,104
	White	164,366	1,000	161,605	486,471
	Green	53,027	1,000	52,404	52,497
**Material**	PLA	384,966	1,000	378,996	764,847
	ABS	271,182	1,000	266,150	644,241
	PETG	220,916	1,000	214,965	545,784
**Material-Color**	PLA Red	55,173	1,000	54,195	161,662
	PLA Yellow	53,782	1,000	51,544	52,172
	PLA Black	56,216	1,000	54,422	112,548
	PLA Magenta	54,555	1,000	53,859	108,786
	PLA Blue	56,858	1,000	55,757	111,534
	PLA Transparent	54,502	1,000	55,804	55,645
	PLA White	53,880	1,000	53,415	162,500
	ABS Red	54,801	1,000	52,926	159,378
	ABS Magenta	53,569	1,000	52,789	107,846
	ABS Blue	54,287	1,000	53,562	107,687
	ABS White	55,498	1,000	54,469	216,833
	ABS Green	53,027	1,000	52,404	52,497
	PET Red	53,536	1,000	52,487	159,243
	PET Black	56,868	1,000	54,901	167,944
	PET Transparent	55,524	1,000	53,856	111,459
	PET White	54,988	1,000	53,721	107,138

Table S9. Number of pixels labeled from the *HSI plastic dataset* in training, validation, and test sets divided into *color*, *material*, and *material-color*. Original training set contain all pixels labeled before applied data reduction. Reduced training set contain the pixels used to train the supervised classifier. ABS: Acrylonitrile Butadiene Styrene; PLA: Polylactic Acid; PETG: Polyethylene Terephthalate Glycol.

Supplementary Results

VNIR-NIR Spectral Fusion Methods Evaluation

K-means algorithm and Support Vector Machine (SVM) classifier were employed to perform the evaluation of the fusion performance comparing the results before and after the proposed fusion procedure using the *HSI plastic dataset*. Hyperparameter optimization of the SVM classifier was performed and the results are shown in Table S10 in the Supplementary Material. Three data fusion methods were compared in this experiment: the fused data (F-O), the fused data without applying the reflectance offset adjustment (F-C), and the fused data without applying band removing and the reflectance offset adjustment (only removing the overlapped spectral region) (F-CNoBR). The results are presented in Fig. S14 and S15 in the Supplementary Material. The results obtained in the segmentation improve without applying the offset adjustment, and, while similar results were obtained in the classification. In addition, Table S11 and S12 in the Supplementary Material details the Jaccard and accuracy results obtained with K-means and SVM for each test HS image and their average and standard deviation values. These results indicate that the F-C and F-C-NoBR methods performs slightly better than the proposed method where the reflectance offset adjustment is applied. However, some of these differences were not statistically significant and could depend on the type of samples to be captured and analyzed by the HS system.

Classifier	HP	Fusion Method	Method	Coarse Search I/S/F	Coarse Search Optimal	Fine Search I/S/F	Fine Search Optimal	Accuracy (%)
SVM-Linear	C	F-O	Color	$2^{-20}/2^{2}/2^{60}$	2^{8}	$2^{6}/2^{0.5}/2^{10}$	2^{7}	89.15
			Material	$2^{-20}/2^{2}/2^{60}$	2^{-2}	$2^{-4}/2^{0.5}/2^{0}$	2^{-3}	83.56
			Material-Color	$2^{-20}/2^{2}/2^{60}$	2^{4}	$2^{2}/2^{0.5}/2^{6}$	2^{5}	82.65
SVM-Linear	C	F-C	Color	$2^{-6}/2^{2}/2^{10}$	2^{0}	$2^{-2}/2^{0.5}/2^{2}$	$2^{0.5}$	90.40
			Material	$2^{-6}/2^{2}/2^{10}$	2^{2}	$2^{0}/2^{0.5}/2^{4}$	2^{3}	91.66
			Material-Color	$2^{-6}/2^{2}/2^{10}$	2^{-2}	$2^{-4}/2^{0.5}/2^{0}$	2^{-4}	**85.54**
SVM-Linear	C	F-C-noBR	Color	$2^{-6}/2^{2}/2^{10}$	2^{2}	$2^{0}/2^{0.5}/2^{4}$	2^{2}	**90.79**
			Material	$2^{-6}/2^{2}/2^{10}$	2^{4}	$2^{2}/2^{0.5}/2^{6}$	2^{4}	**91.93**
			Material-Color	$2^{-6}/2^{2}/2^{10}$	2^{2}	$2^{0}/2^{0.5}/2^{4}$	2^{2}	84.23

Table S10. Coarse-to-fine search to optimize the Cost (C) hyperparameter of the SVM classifier using the three different fusion methods with the validation set of the *HSI plastic dataset*. F-O: Fused data applying reflectance offset adjustment; F-C: Fused data without applying reflectance offset adjustment; F-C-NoBR: Fused data without applying reflectance offset adjustment and band removing; HP: Hyperparameter; I: Initial value; S: Step value; F: Final value

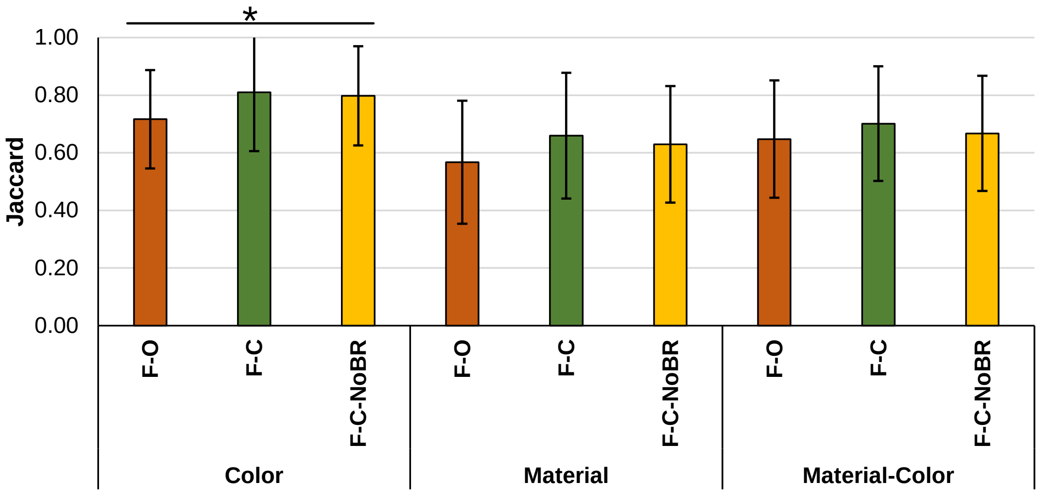

Figure S14. Average Jaccard results obtained with the entire test set from the *HSI plastic dataset* using the K-means algorithm for the three different fusion methods. Results were statistically analyzed using a paired, one-tailed Student's T-test at the 5% significance level. (*) Statistically significant difference ($p < 0.05$). F-O: Fused data applying reflectance offset adjustment; F-C: Fused data without applying reflectance offset adjustment; F-C-NoBR: Fused data without applying reflectance offset adjustment and band removing.

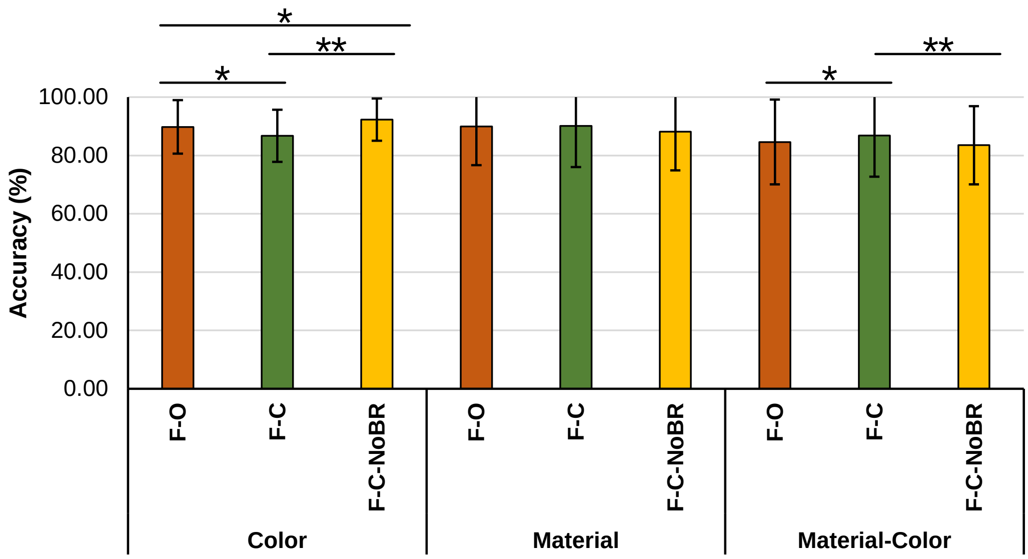

Figure S15. Average accuracy results obtained with the entire test set from the *HSI plastic dataset* using the SVM classifier for the three different fusion methods. Results were statistically analyzed using a paired, one-tailed Student's T-test at the 5% significance level. (*) Statistically significant difference ($p < 0.05$). (**) Highly statistically significant difference ($p < 0.001$). F-O: Fused data applying reflectance offset adjustment; F-C: Fused data without applying reflectance offset adjustment; F-C-NoBR: Fused data without applying reflectance offset adjustment and band removing.

	Color			Material			Material-Color		
	F-O	F-C	F-C-noBR	F-O	F-C	F-C-noBR	F-O	F-C	F-C-noBR
**R1**	0.77	**0.97**	0.95	0.41	**0.91**	0.45	0.41	**0.51**	0.45
**R2**	**0.87**	0.61	0.81	0.32	0.57	**0.70**	0.42	0.57	**0.70**
**R3**	0.52	0.39	**0.77**	0.34	**0.74**	0.43	0.51	**0.74**	0.43
**R4**	**0.51**	0.41	0.41	0.31	**0.52**	0.38	0.31	0.32	**0.38**
**R5**	0.94	**0.96**	0.93	0.73	**0.95**	0.56	**0.73**	0.49	0.56
**R6**	0.69	**0.95**	0.92	**0.48**	0.41	0.43	0.48	**0.95**	0.43
**R7**	0.63	**0.87**	0.84	0.55	**0.65**	0.64	0.63	**0.87**	0.84
**R8**	0.48	**0.95**	0.51	0.63	0.40	**0.92**	0.90	0.53	**0.93**
**R9**	0.70	**0.86**	0.66	0.56	0.40	**0.63**	0.70	**0.86**	0.66
**R10**	0.87	**0.91**	0.88	0.87	**0.91**	0.88	0.87	**0.91**	0.88
**R11**	0.52	0.82	**0.87**	**0.89**	0.82	0.87	**0.89**	0.82	0.87
**R12**	0.87	**0.88**	0.87	0.87	**0.88**	0.87	0.87	**0.88**	0.87
**R13**	0.94	**0.96**	0.95	0.42	**0.43**	0.42	**0.70**	0.67	0.68
**Avg.**	0.72	**0.81**	0.80	0.57	**0.66**	0.63	0.65	**0.70**	0.67
**Std.**	**0.17**	0.20	**0.17**	0.21	0.22	**0.20**	**0.20**	**0.20**	**0.20**

Table S11. Jaccard results for the K-means algorithm of each test HS image from the *HSI plastic dataset* and their average (avg) and standard deviation (std). The results are divided into *color*, *material*, and *material-color* segmentation problems, using the three different fusion methods. F-O: Fused data applying reflectance offset adjustment; F-C: Fused data without applying reflectance offset adjustment; F-C-NoBR: Fused data without applying reflectance offset adjustment and band removing.

	Color			Material			Material-Color		
	**F-O**	**F-C**	**F-C-noBR**	**F-O**	**F-C**	**F-C-noBR**	**F-O**	**F-C**	**F-C-noBR**
**R1 (%)**	**97.0**	94.7	95.8	97.4	**98.2**	96.0	94.2	**97.1**	96.2
**R2 (%)**	82.2	89.5	**95.9**	47.9	46.2	**49.8**	44.9	47.8	**49.7**
**R3 (%)**	72.0	71.8	**76.3**	95.4	**96.5**	95.4	72.1	**78.5**	73.5
**R4 (%)**	76.9	72.5	**82.5**	**92.8**	89.8	88.2	**95.1**	93.4	88.8
**R5 (%)**	93.9	93.0	**97.1**	93.3	**96.6**	96.3	93.1	**96.6**	96.2
**R6 (%)**	96.1	**96.9**	96.7	96.0	**98.2**	97.7	94.9	**97.7**	97.1
**R7 (%)**	77.2	73.8	**80.9**	98.2	**98.4**	97.6	74.4	**79.7**	79.0
**R8 (%)**	**96.6**	88.8	96.2	**96.9**	96.2	81.7	91.9	**97.0**	77.8
**R9 (%)**	92.8	92.1	**96.3**	97.1	**97.5**	96.6	94.9	**96.3**	95.2
**R10 (%)**	96.4	87.2	**96.5**	85.0	**87.1**	77.7	85.2	**89.1**	77.8
**R11 (%)**	92.3	82.4	**93.4**	90.5	88.2	**91.6**	81.8	79.3	**83.9**
**R12 (%)**	96.5	88.3	**96.8**	**88.4**	84.1	83.4	**80.9**	80.5	76.7
**R13 (%)**	**97.4**	96.7	95.7	90.3	94.6	**94.7**	96.5	**96.1**	93.8
**Avg. (%)**	89.8	86.7	**92.3**	89.9	**90.1**	88.2	84.6	**86.9**	83.5
**Std. (%)**	9.2	9.0	**7.3**	**13.2**	14.1	13.3	14.5	14.1	**13.4**

Table S12. Accuracy results for the SVM classification of each test HS image from the HSI plastic dataset and their average (avg) and standard deviation (std). The results are divided into color, material, and material-color segmentation problems, using the three different fusion methods. F-O: Fused data applying reflectance offset adjustment; F-C: Fused data without applying reflectance offset adjustment; F-C-NoBR: Fused data without applying reflectance offset adjustment and band removing.

Supplementary Methods

HS Database

The HSI registration dataset was composed by seven HS images obtained from four different spatial patterns, i.e., a shooting target and patterns based on brain morphological structures (Fig. S1a). The *HSI spectral reference dataset* (Fig. S1b) was composed by six HS images obtained from three different Spectralon White Diffuse Reflectance Standards (Labsphere Inc., North Sutton, US) with a reflectance value of 99%, where two consecutive captures were obtained from each white reference. In addition, an HS image from a Zenith Polymer Reflectance Standard (SphereOptics GmbH, Germany), composed by rare earth oxides, was captured (SR4 in Fig. S1b). The *HSI plastic dataset* was composed by different samples of 3D printing filament, as polylactic acid (PLA), acrylonitrile butadiene styrene (ABS), and polyethylene terephthalate glycol (PETG). Using the Ultimaker 3 Extended (Utrecht, Netherlands) 3D printer, several 32 × 32 × 4.8 mm square samples were printed to create the *HSI plastic dataset*. In addition to the three different materials, different colors were also printed. At the end, twenty HS images from sixteen different plastic samples were obtained (Fig. S1c). *HSI plastic dataset* was partitioned into training (four HS images), validation (three HS images) and test (thirteen HS images) sets. Additionally, the dataset was organized into three groups for performing different classification and segmentation problems: *color*, *material*, and *material-color*. Then, each sample was labelled in different classes corresponding to each problem type. The labelled pixels were divided into training, validation, and test set. The training set was reduced using a methodology based on K-Means. The goal of this methodology is to reduce the number of pixels in each class, avoiding the inclusion of redundant information, and drastically reducing the training execution time. The K-means was applied to each class each class of the twenty-seven classes contained in the dataset, obtaining a total of 1000 pixels per class, reducing the total number of pixels from 2,631,192 to 27,000. This methodology was used to reduce and balance the training set employed in supervised classifier to identify brain cancer[1]. Finally, the *HSI brain dataset* (Fig. S1d) is formed by three HS images of in vivo brain tissue acquired at the University Hospital of Gran Canaria Doctor Negrin, Spain. Written informed consent was obtained from all participant subjects, and the study protocol and consent procedures were approved by the *Etica de la Investigacion / Comite de Etica de la Investigacion con Medicamentos (DEI/CEIM)* of the University Hospital Doctor Negrin (2019-001-1). This dataset is employed to perform a preliminary evaluation of the proposed configuration of the acquisition system and the proposed VNIR-NIR fusion approach in a real environment. These HS images were previously labeled into two classes (*normal tissue* and *blood vessels)* using a semiautomatic Labelling Tool[2] based on Spectral Angle Mapper (SAM) algorithm to obtain the ground-truth maps.

Data pre-processing

The HS image was calibrated following Eq. (S1), where CI is the calibrated image, RI is the raw image, and WI and DI are the white and dark reference images, respectively. The white reference image was acquired, in the same illumination conditions that the image was captured, using a standard white reference tile that reflects the 99% of the incident light. The dark reference image was obtained by keeping the camera shutter closed, being used to avoid the dark currents produced by the camera sensor. Finally, a data smoothing approach based on a moving average filter was applied for reducing the high-frequency noise. Each smoothed value was averaged using a window of five data points.

$$CI = \frac{RI - DI}{WI - DI} \quad \text{(S1)}$$

Additionally, due to both HS cameras have different spatial resolutions, it was necessary to resample one of the two HS images to be able to register them. The VNIR camera covered the spectral range between 400 and 1000 nm, can capture 1004 spatial pixels with a pixel pitch of 7.4 µm, while the NIR camera captured information within the 900-1700 nm spectral range, 320 spatial pixels and a pixel pitch of 30 µm. The lens used in VNIR camera was a Xenoplan 1.4 (Schneider Optics, Hauppauge, NY, USA) with a focal length of 23 mm and a working distance of ~42 cm. In the NIR camera, the lens used was a Kowa LM25HC-SW 1.4 (Kowa Optimed Deutschland GmbH, Düsseldorf, Germany) with 25 mm of focal length and a working distance of ~33 cm.

Upsampling and downsampling methods were evaluated to achieve the same spatial resolutions in both HS images. To exploit the increased VNIR spatial resolution for a later visualization and manual labeling of the images with high detail in the targeting application (intraoperative HS brain cancer detection), the downsampling method where the VNIR spatial resolution is reduced to reach the NIR pixels size was discarded. In this targeted application, to generate a labelled dataset, the brain images must be manually labeled, identifying the different classes (tumor, normal and hypervascularized tissue). The high VNIR spatial resolution and the possibility of generating a RGB image allows neurosurgeons to visualize the different brain regions and identify the relevant pixels to be labeled using a semi-automatic labeling tool developed to this end[3]. However, the low spatial resolution in the NIR camera was not enough for performing a reliable labeling of the brain surface. For this reason, the spatial resolution of the NIR image was upsampled until reaching the VNIR pixel size.

The scale factor to perform the spatial resampling is specified by the relation between the instantaneous field of view (IFOV) of both cameras. The IFOV is calculated employing the parameters of each camera following Eq. (S2), achieving an IFOV value of 0.402 and 0.137 nm for the NIR and VNIR cameras, respectively. Hence, a scale factor of 293.43% was obtained following Eq. (S3). This scale factor remains the same independently of the sample type, as long as the HS acquisition system does not suffer any modification. Hence, the HS images have a fixed width and height in both HS cameras with a fixed working distance of ~33 and ~42 cm in NIR and VNIR cameras, respectively. However, if there is any modification in the system, such as different HS cameras, lenses, distance between the HS cameras or the working distance, the scale factor must be recalculated with the new parameters.

$$IFOV\ (mm) = \frac{Pixel\ Pitch \cdot Working\ Distance}{Focal\ Length} \quad \text{(S2)}$$

$$Scale\ Factor\ (\%) = \frac{IFOV_{NIR}}{IFOV_{VNIR}} \quad \text{(S3)}$$

The upsampling algorithm used to increase the NIR spatial resolution (from 320 × 253 to 939 × 743 pixels) and to estimate the upsampled spectral signatures is based on a bilinear interpolation, considering the nearest 2-by-2 neighborhood of a certain pixel. Fig. S13 in the Supplementary Material shows a graphical representation of this methodology. Nearest-neighbor, bilinear, and bicubic interpolation methods were evaluated using the SAM algorithm. To perform this evaluation, a region of 15 × 15 pixels was selected from four different reference materials (Zenith Polymer Reflectance Standard, and three different plastics [PLA, ABS, and PETG]). The SAM algorithm was computed using the mean spectral signature before and after the interpolation. Table S13 in the Supplementary Material shows the SAM results obtained with each interpolation method and the corresponding execution time. It can be observed that all methods have similar SAM values, however, bilinear interpolation method achieved the lowest, followed by the nearest-neighbor and bicubic methods. On the contrary, the nearest-neighbor interpolation offered the lowest execution time, followed by the bicubic and bilinear methods.

	**Interpolation Method**					
	**Nearest-neighbor**		**Bilinear**		**Bicubic**	
	**SAM**	**Time (ms)**	**SAM**	**Time (ms)**	**SAM**	**Time (ms)**
**Polymer**	3.02e-04	**1.80**	**2.86e-04**	3.70	4.91e-04	3.15
**PLA**	5.98e-04	**1.93**	**5.83e-04**	3.54	6.61e-04	2.91
**ABS**	3.98e-04	**2.22**	**2.76e-04**	3.65	2.98e-04	3.01
**PETG**	7.33e-04	**2.01**	**5.17e-04**	3.76	5.54e-04	2.98

Table S13. Spectral Angle Mapper (SAM) and execution time results of different interpolation methods for the evaluation. The SAM algorithm was computed using the mean spectral signature of a region of 15 × 15 before and after the interpolation. Four different reference materials (Zenith Polymer Reflectance Standard, and three different plastics [PLA, ABS, and PETG]) were employed in this evaluation. PLA: Polylactic Acid; ABS: Acrylonitrile Butadiene Styrene: PETG: Polyethylene Terephthalate Glycol.

VNIR-NIR spatial registration

In this study, intensity-based and feature-based techniques were employed for registering the VNIR and NIR images. MATLAB Image Processing Toolbox and Computer Vision Toolbox (The MathWorks Inc., Natick, MA, USA) was employed to implement the registration algorithms. On the one hand, intensity-based techniques find the maximum (or minimum) intensity value and correlate it with the intensity value of the reference image to transform the misaligned image. To obtain the maximum (or minimum) intensity value an iterative process is performed where the parameters of the transformation model are modified in each iteration. An evolutionary optimizer was used to find the best geometric transformation model. The intensity value is measured applying Mattes Mutual Information (MMI)[4]. The geometric transform types evaluated in this technique were: 1) Translation transformations, where each pixel is displaced the same amount in the same direction, but the size and orientation are not modified; 2) Similarity transformations, which preserve shape, but not size, including isotropic scaling, rotation, and translation; and 3) Affine transformations, which include all similarity transformations and also preserve parallel lines.

On the other hand, feature-based techniques detect and extract interest points, curves, or surfaces present in both images without consider the image intensity. The number of common features detected in both images must be enough to perform the registration, determining the quality results[5]. The feature-based techniques are composed by several steps: 1) Feature detection and extraction, where regions, lines, and curves are detected using feature detectors and subsequently extracted using feature extractors; 2) Feature matching, where all possible matching points between both images are found using an exhaustive matching method computing the pair-wise distance between features; 3) Geometric transform estimation, where different transformation types can be used (similarity, affine or projective). The projective transformation includes all affine transformation and also supports tilting. The matching pair are used to estimate the transformation matrix and the m-estimator sample consensus (MSAC) algorithm is used to exclude outlier points[6]. This algorithm has a randomized nature and can offers different results in consecutive executions.

In this work, two feature detectors and extractors were evaluated: speeded up robust features (SURF)[7] and maximally stable extremal regions (MSER)[8]. SURF is a fast algorithm based on the Hessian-Matrix to find the keypoints. SURF has been used to detect skin features to track patient position in navigated spinal surgery[9]. On the contrary, MSER algorithm is a shape-based method that detects regions using image intensity. Regions where the intensity values do not change are considered maximally stable. MSER has been used in the literature as shape detector in medical applications to segment cells[10]. Also, SURF and MSER algorithms were selected there are robust to changes in scale and rotation.

To evaluate the performance of the different registration techniques and geometric transformations, a gray-scale image was generated from a pseudo-RGB image of both VNIR and NIR HS cubes for performing the registration. After selecting the registration techniques and geometric transformations, the transformation matrix was obtained comparing a pair of VNIR and NIR spectral bands. The pseudo-RGB images were generated selecting three bands which correspond with red, green, and blue colors in the VNIR range and other three bands for creating a false-color RGB in the NIR range (see Fig. 6c and d). The wavelengths selected for VNIR pseudo-RGB image were 708.97 nm (red), 539.44 nm (green), and 479.06 nm (blue). The wavelengths selected for NIR pseudo-RGB image were 1094.89 nm (red), 1247.44 nm (green), and 1595.45 nm (blue). These wavelengths were selected to maintain the compatibility with the original software (Hyperspec III software, Headwall Photonics Inc., Fitchburg, MA, USA) provided by the camera manufacturer. Additionally, these wavelengths were employed in previous works for generating the NIR pseudo-RGB image[11]).

VNIR-NIR spectral fusion

This section explains the procedure followed to obtain the reflectance offset employed to adjust the NIR spectral signature within the proposed fusion method. This procedure requires the use of the NIR image ($N \in \mathbb{R}^P \otimes \mathbb{R}^B$) and the VNIR image ($V \in \mathbb{R}^P \otimes \mathbb{R}^B$) of the captured scene, as well as the certified spectral signature provided by the manufacturer of the Zenith Polymer Reflectance Standard ($P \in \mathbb{R}^B$). B represents the number of spectral bands in each HS image and P the number of pixels of the corresponding HS image. The procedure is as follows. First, the difference in the reflectance levels in the Zenith Polymer (P_{Offset}) is computed following Eq. (S4), where the value corresponding with the first spectral band of the NIR image ($\lambda_{NIR} = 956\,nm$) is $P(\lambda_{NIR})$ and the value corresponding with the last spectral band of the VNIR image ($\lambda_{VNIR} = 901\,nm$) is $P(\lambda_{VNIR})$. The manufacturer provided the certified data in percentage format. Fig. S16a shows a graphical representation of this first step. Next, the average value of all pixels in the last band of the VNIR image ($\bar{V}_{\lambda_{VNIR}}$) and the first band of the NIR image ($\bar{N}_{\lambda_{NIR}}$) are computed as expressed in Eq. (S5). The difference of these two values (Fig. S16b) is employed to obtain the reflectance offset value (F_{Offset}) for the captured scene following Eq. (S6). Finally, this offset is applied to each pixel of the NIR image from the captured scene independently, resulting in the fused spectra as show in Fig. S16c.

$$P_{Offset}(\%) \ = P(\lambda_{NIR}) - P(\lambda_{VNIR}) \tag{S4}$$

$$\bar{V}_{\lambda_{VNIR}} = \sum_{i=1}^{P} \frac{V(i,\lambda_{VNIR})}{P}; \ \bar{N}_{\lambda_{NIR}} = \sum_{i=1}^{P} \frac{N(i,\lambda_{NIR})}{P} \tag{S5}$$

$$F_{Offset} = \left(\bar{V}_{\lambda_{VNIR}} - \bar{N}_{\lambda_{NIR}}\right) \cdot (P_{Offset} + 1) \tag{S6}$$

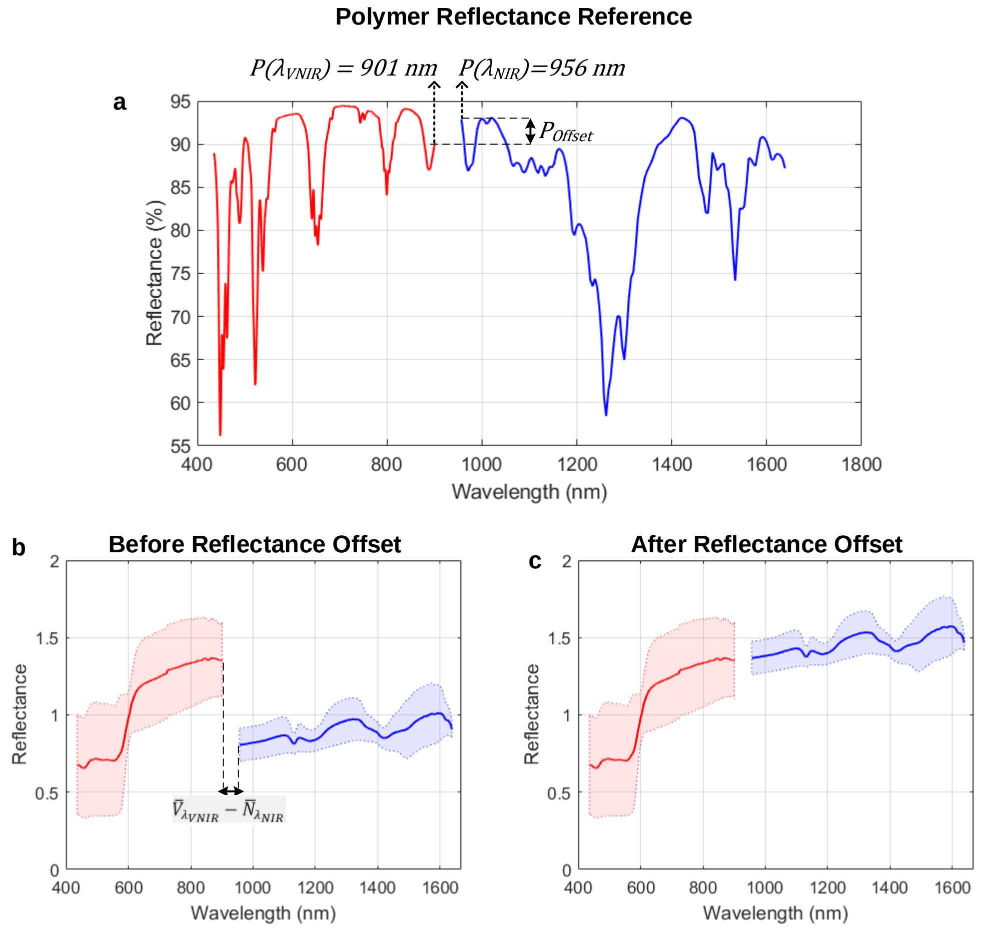

Figure S16. Proposed VNIR-NIR spectral fusion explanation using, as example, one sample of the *HSI plastic dataset*. (**a**) Zenith Polymer Reflectance Standard spectrum provided by the manufacturer representing the P_{Offset} constant used to perform the spectral fusion. (b) Average and standard deviation of the spectral signatures extracted from the plastic sample before applying the reflectance offset adjustment to the NIR data. (c) Average and standard deviation of the spectral signatures extracted from the plastic sample after applying the reflectance offset adjustment to the NIR data. $P(\lambda_{NIR})$: Pixel value corresponding with the first spectral band of the NIR image in the Zenith Polymer Reflectance Standard spectrum provided by the manufacturer ($\lambda_{NIR} = 956\,nm$); $P(\lambda_{VNIR})$: Pixel value corresponding with the last spectral band of the VNIR image in the Zenith Polymer Reflectance Standard spectrum provided by the manufacturer ($\lambda_{VNIR} = 901\,nm$); $\bar{V}_{\lambda_{VNIR}}$: Average value of all pixels in the last band of the VNIR image of the captured scene; $\bar{N}_{\lambda_{NIR}}$: Average value of all pixels in the first band of the NIR image of the captured scene.

Segmentation and Classification Methods

The segmentation method employed the K-means, K-medoids, and hierarchical K-means algorithms. K-means and K-medoids are similar clustering algorithms widely used to segment HS images into K different clusters[2]. K-medoids is robust to outliers and the centroid of each cluster is an actual spectrum found in the cluster set, while in the K-means algorithm, the cluster centroid is the average value of all spectra in the cluster set[12]. Hierarchical clustering organizes the data into a tree structure, being the number of trees defined by the K value. Hierarchical K-means uses K-means to split the clusters[13]. The number of clusters (K) was previously selected and, in the case of HSI plastic dataset, the selected K value corresponds to the number of classes present in the ground-truth of each HS image to be processed. In the case of HSI brain dataset, the number of clusters used was twenty-four. This number was selected based on the results of a previous work[2]. Finally, to obtain the segmentation maps, the clusters more similar to the ground-truth were selected using Jaccard metric. In these experiments, the clusters initialization was performed using the same seed. K-means and hierarchical K-means algorithms have been used for HS data segmentation to identify brain cancer[2,14]. MATLAB Statistics and Machine Learning Toolbox (The MathWorks Inc., Natick, MA, USA) was employed to implement the K-means algorithms.

The pixel-wise supervised classification was based on the SVM, Random Forest (RF), and K-Nearest Neighbors (KNN) classifiers. The SVM classifier has the objective of separating different data by finding out the best hyperplane with a maximum margin, being widely used for classification and regression purposes[15]. In this study, a linear hyperplane was used to separate the data applying a linear kernel. The RF classifier is based on decision trees, identifying the new data class by taking a vote of their predictions from an aggregation of decision trees[16]. Finally, the KNN classifier compares each incoming sample with all their neighbors using a distance metric to find the closest neighbors[17]. In this work, the cosine distance metric was employed. In the classification problem, the HSI plastic dataset was partitioned into training, validation, and test sets. The training and validation sets were used to optimize, evaluate, and generate the classification model. After the hyperparameter optimization, the performance of the model was evaluated using the test set. These algorithms have been widely used to identify glioblastoma tumor in pathological slides and in-vivo tissue using HS data[18,19]. The LIBSVM library was used as SVM implementation[20], while the MATLAB Statistics and Machine Learning ToolBox was employed for the RF and KNN implementations.

Performance Metrics

The spatial registration was evaluated using image-based similarity and overlap-based metrics. Mutual Information (MI) measures the dependency between two images X and Y[21]. This can be expressed as Eq. (S7), where $p_X(x)$ and $p_Y(y)$ are the marginal probability distributions of X and Y, respectively, and $p_{XY}(x,y)$ denotes the joint probability distribution of X and Y. When the optimal alignment occurs, the MI is maximized. The Pearson's Correlation Coefficient (PCC) is widely used for comparing images[22]. This coefficient measures the degree of linear correlation or anti-correlation between two sets of data in the range [-1,1], where $PCC = -1$ indicates perfectly anti-correlated images, $PCC = 1$ indicates perfectly linearly correlated images, and $PCC = 0$ indicates linearly uncorrelated images. The PCC can be expressed as the covariance between two images by the product of their standard deviations (Eq. (S8)). The Structural Similarity Index measure (SSIM) is a metric commonly used in image compression to evaluate the compressed image against the original uncompressed image[23]. SSIM metric is computed considering the luminance, contrast and structure terms as shown in Eq. S9-11), where μ and σ represent the mean and standard deviations for x and y, and σ_{xy} represents the cross-covariance for x, y. Combining the three terms, SSIM can be expressed as show in Eq. (S12) with an index range [-1,1], where $SSIM = -1$ indicate uncorrelated images and $SSIM = 1$ indicates correlated images.

$$MI(X;Y) = \sum_{x,y} p_{X,Y}(x,y) \log \frac{p_{XY}(x,y)}{p_X(x)p_Y(y)} \tag{S7}$$

$$PCC = \frac{cov(X,Y)}{\sigma_X \sigma_Y} \tag{S8}$$

$$l(x,y) = \frac{2\mu_x\mu_y + C_1}{\mu_x^2 + \mu_y^2 + C_1} \tag{S9}$$

$$c(x,y) = \frac{2\sigma_x\sigma_y + C_2}{\sigma_x^2 + \sigma_y^2 + C_2} \tag{S10}$$

$$s(x,y) = \frac{\sigma_{xy} + C_3}{2\sigma_x\sigma_y + C_3} \tag{S11}$$

$$SSIM(x,y) = [l(x,y)]^{\alpha} \cdot [c(x,y)]^{\beta} \cdot [s(x,y)]^{\gamma} \tag{S12}$$

In order to measure the spectral repeatability of the acquisition system and to evaluate the signal-to-noise ratio in each spectral band, the absolute relative difference percentage (RD) metric was employed. This metric computes the relation between the absolute difference and the mean values of two vectors following Eq. (S13). A lower RD value in a certain spectral band implies lower differences between the two bands of the same scene, therefore, better repeatability of the acquisition system and higher signal-to-noise ratio. This metric has been previously employed to evaluate the repeatability of the original intraoperative HS acquisition system[3].

$$RD\ (\%) = \frac{abs(x-y) \cdot 100}{(mean(x) + mean(y))/2} \tag{S13}$$

The segmentation problem performance was evaluated using overlap-based metrics. After applying the K-means algorithm to VNIR, NIR and fused images, the ground-truth (*GT*) and the segmentation image (*SI*) were evaluated using the Jaccard similarity coefficient, which measures the similarity between two images and it is defined as the intersection over the union of two images (Eq. (S14))[24]. This metric has a value range in [0, 1], and it can be expressed using the definition related with of true positives (*TP*), false positives (*FP*), and false negatives (*FN*).

$$Jaccard = \frac{|GT \cap SI|}{|GT \cup SI|} = \frac{TP}{TP + FP + FN} \tag{S14}$$

Finally, the classification problem was evaluated using the accuracy metric defined by Eq. (S15), where TN represent the number of true negatives.

$$Accuracy = \frac{TP + TN}{TP + TN + FP + FN} \tag{S15}$$

Additionally, segmentation and classification results were statistically analyzed using a paired, one-tailed Student's T-test at the 5% significance level.

Supplementary References

1. Martinez, B. *et al.* Most Relevant Spectral Bands Identification for Brain Cancer Detection Using Hyperspectral Imaging. *Sensors* **19**, 5481 (2019).
2. Fabelo, H. *et al.* Spatio-spectral classification of hyperspectral images for brain cancer detection during surgical operations. *PLoS One* **13**, 1–27 (2018).
3. Fabelo, H. *et al.* In-Vivo Hyperspectral Human Brain Image Database for Brain Cancer Detection. *IEEE Access* **7**, 39098–39116 (2019).
4. Mattes, D., Haynor, D. R., Vesselle, H., Lewellyn, T. K. & Eubank, W. Nonrigid multimodality image registration. in *Medical Imaging 2001: Image Processing* (eds. Sonka, M. & Hanson, K. M.) **4322**, 1609–1620 (SPIE, 2001).
5. Zitová, B. & Flusser, J. Image registration methods: A survey. *Image Vis. Comput.* **21**, 977–1000 (2003).
6. Torr, P. H. S. & Zisserman, A. MLESAC: A new robust estimator with application to estimating image geometry. *Comput. Vis. Image Underst.* **78**, 138–156 (2000).
7. Bay, H., Tuytelaars, T. & Van Gool, L. SURF: Speeded up robust features. in *Lecture Notes in Computer Science (including subseries Lecture Notes in Artificial Intelligence and Lecture Notes in Bioinformatics)* **3951 LNCS**, 404–417 (Springer, Berlin, Heidelberg, 2006).
8. Matas, J., Chum, O., Urban, M. & Pajdla, T. Robust wide-baseline stereo from maximally stable extremal regions. in *Image and Vision Computing* **22**, 761–767 (Elsevier Ltd, 2004).
9. Manni, F. *et al.* Hyperspectral Imaging for Skin Feature Detection: Advances in Markerless Tracking for Spine Surgery. *Appl. Sci.* **10**, 4078 (2020).
10. Krig, S. & Krig, S. Interest Point Detector and Feature Descriptor Survey. in *Computer Vision Metrics* 217–282 (Apress, 2014). doi:10.1007/978-1-4302-5930-5_6
11. Fabelo, H. *et al.* An Intraoperative Visualization System Using Hyperspectral Imaging to Aid in Brain Tumor Delineation. *Sensors* **18**, 430 (2018).
12. Massoud, M. A. & Kaldas, M. M. Comparative Study of Hyperspectral Partitioning Clustering Algorithms for Mineral Exploration. *Minia J. Eng. Technol.* **38**, (2019).
13. Gillis, N., Kuang, D. & Park, H. Hierarchical Clustering of Hyperspectral Images using Rank-Two Nonnegative Matrix Factorization.
14. Torti, E. *et al.* Parallel K-Means Clustering for Brain Cancer Detection Using Hyperspectral Images. *Electronics* **7**, 283 (2018).
15. Ortega, S. *et al.* Detecting brain tumor in pathological slides using hyperspectral imaging. *Biomed. Opt. Express* **9**, 818 (2018).
16. Dietterich, T. G. Ensemble Methods in Machine Learning. in *Multiple Classifier Systems* 1–15 (Springer Nature, 2000). doi:10.1007/3-540-45014-9_1
17. Huang, K., Li, S., Kang, X. & Fang, L. Spectral–Spatial Hyperspectral Image Classification Based on KNN. *Sens. Imaging* **17**, 1–13 (2016).
18. Ortega, S. *et al.* Hyperspectral Imaging for the Detection of Glioblastoma Tumor Cells in H&E Slides Using Convolutional Neural Networks. *Sensors* **20**, 1911 (2020).
19. Fabelo, H. *et al.* Surgical aid visualization system for glioblastoma tumor identification based on deep learning and in-vivo hyperspectral images of human patients. in *Medical Imaging 2019: Image-Guided Procedures, Robotic Interventions, and Modeling* (eds. Fei, B. & Linte, C. A.) **10951**, 35 (SPIE, 2019).
20. Chang, C. & Lin, C. LIBSVM : A Library for Support Vector Machines. *ACM Trans. Intell. Syst. Technol.* **2**, 1–39 (2013).
21. Wells Iii, W. M., Viola, P., Atsumi, H., Nakajima, S. & Kikinis, R. *Multi-modal volume registration by maximization of mutual information*. *Medical Image Analysis* **1**, (1996).
22. Pearson, K. Mathematical Contributions to the Theory of Evolution. III. Regression, Heredity, and Panmixia. *Philos. Trans. R. Soc. A Math. Phys. Eng. Sci.* **187**, 253–318 (1896).
23. Wang, Z., Bovik, A. C., Sheikh, H. R. & Simoncelli, E. P. Image quality assessment: From error visibility to structural similarity. *IEEE Trans. Image Process.* **13**, 600–612 (2004).
24. Jaccard, P. Étude comparative de la distribution florale dans une portion des Alpes et des Jura. *Bull. del la Société Vaudoise des Sci. Nat.* **37**, 547–579 (1901).
